# Transition From Land to Sea: Comparative Genomics Illuminates the Adaptive Evolution of the Intertidal Spider

**DOI:** 10.1111/1755-0998.70147

**Published:** 2026-04-27

**Authors:** Fan Li, Yunyun Lv, Chao Bian, Quentin Moana Perrin, Yubo Zhang, Yixiao Wang, Lei Cao, Long Yu, Shengtao Guo, Wei Zhang, Qiong Shi, Daiqin Li

**Affiliations:** ^1^ Centre for Behavioural Ecology and Evolution, School of Life Sciences Hubei University Wuhan China; ^2^ State Key Laboratory of Gene Function and Modulation Research, School of Life Sciences Peking University Beijing China; ^3^ Laboratory of Sichuan Province for Fishes Conservation and Utilization in the Upper Reaches of the Yangtze River, College of Life Sciences Neijiang Normal University Neijiang China; ^4^ Laboratory of Aquatic Genomics, College of Life Sciences and Oceanography Shenzhen University Shenzhen China; ^5^ Centre for Sustainable Materials, School of Materials Science and Engineering Nanyang Technological University Singapore Singapore; ^6^ Peking‐Tsinghua Center for Life Sciences, Academy for Advanced Interdisciplinary Studies Peking University Beijing China; ^7^ Institute of Ecology Peking University Beijing China; ^8^ Medog Biodiversity Observation and Research Station of Xizang Autonomous Region Nyingchi China

**Keywords:** adaptive evolution, marine environment, osmoregulation, oxygen management, spider silk

## Abstract

Spiders are renowned for their ecological versatility and silk‐based innovations in materials science, yet marine environments remain virtually uncolonized by this predominantly terrestrial lineage. A striking exception is the obligate intertidal spider genus *Desis*, whose members have evolved extraordinary physiological and behavioural adaptations to persist in wave‐swept, saline habitats that oscillate between land and sea. However, the molecular basis of these adaptations has remained largely unexplored. Here, we present a high‐quality, chromosome‐scale genome of the intertidal spider *Desis jiaxiangi*, together with a reference genome of the water spider *Argyroneta aquatica* , integrated with transcriptomic and proteomic data. This multi‐omics framework reveals the genomic architecture underlying adaptation to life at the ocean's edge. We uncover expansions of gene families linked to hormone biosynthesis and DNA repair, alongside signatures of adaptive evolution in genes involved osmoregulation, the rate‐limiting step of glycolysis, mitochondrial regulation, epithelial tube morphogenesis and circadian rhythm. Notably, we characterize a novel silk spidroin enriched with a unique GVGAKV motif, which may enhance silk hydrophobicity, and detect the duplication burst of hemocyanin genes likely supporting oxygen transport during submersion. Together, these findings reveal convergent molecular strategies for coping with extreme and fluctuating environments, and demonstrate how genomic innovation enables terrestrial lineages to invade marine‐influenced ecosystems. Our study establishes *Desis* as powerful model for understanding adaptation at terrestrial–marine interface.

## 1 Introduction

Oceans cover more than 70% of Earth's surface and harbour extraordinary biodiversity, as well as biological innovations crucial for ecosystem stability and biotechnological advancement. Despite their ecological success on land, spiders are rarely associated with marine environments, reflecting their predominantly terrestrial lifestyles and reliance on air‐breathing physiology. On land, spiders play key ecological roles as predators that regulate insect populations (Nyffeler and Birkhofer 2017). Yet, a notable exception to this terrestrial constraint is the spider genus *Desis*, whose members occupy the intertidal zone—one of the most physiologically demanding and ecologically dynamic environments on the planet (Mammola et al. 2017).

**FIGURE 1:**
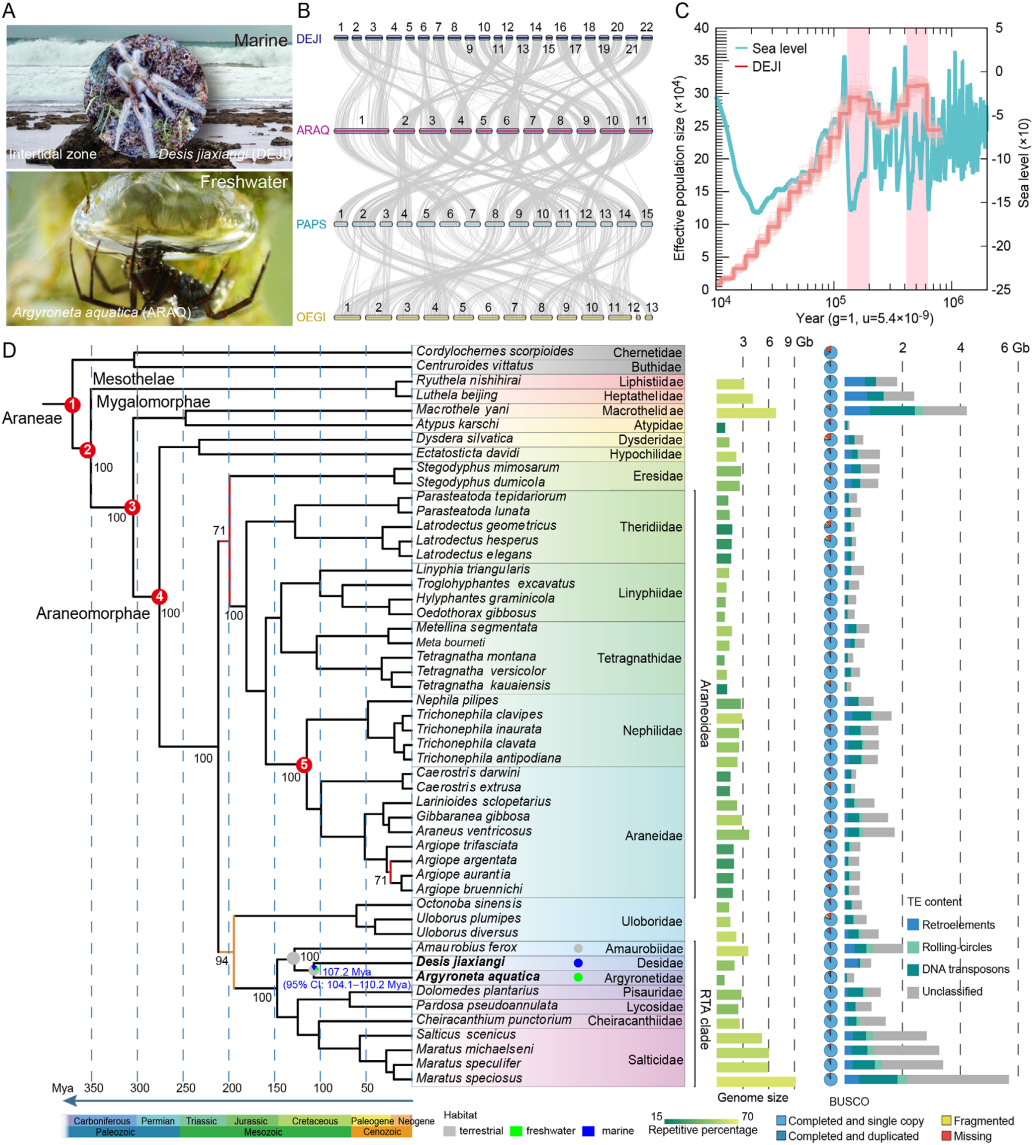
Genomic structure and phylogeny of the intertidal spider *Desis jiaxiangi* and the water spider *Argyroneta aquatica* (A) Photographs of the intertidal spider (centre circle), the water spider with its diving bell and their respective habitats. (B) Synteny comparison among *D. jiaxiangi* (DEJI), *A. aquatica* (ARAQ), *Pardosa pseudoannulata* (PAPS) and *Oedothorax gibbosus* (OEGI). (C) Inferred demographic history of *D. jiaxiangi* based on PSMC analysis. (D) Phylogenomic tree of representative spiders, annotated with genome sizes, repetitive percentages, BUSCO completeness scores and transposable element (TE) contents. Fossil calibrations: Red dots labelled 1–5 denote fossil calibration points used in divergence time estimation. Calibration constraints are as follows: (1) Panscorpiones stem: *Proscorpius osborni* (418–423 Mya); (2) Mesothelae stem: *Palaeothele montceauensis* (299–304 Mya); (3) Avicularioidea stem: *Rosamygale grauvogeli* (242–247.2 Mya); (4) Synspermiata stem: *Eoplectreurys gertschi* (164–175.1 Mya); (5) Nephilinae stem: *Palaeonephila dilitans* (43–47.8 Mya). Nodes without displayed support values have bootstrap support of 100. Ancestral habitat reconstructions for the marronoid spider clade are indicated by icons: Grey (terrestrial), green (freshwater) and blue (marine).

The intertidal zone represents a unique convergence of terrestrial and marine extremes, exposing resident organisms to intense wave action, large and rapid fluctuations in temperature and salinity, high ultraviolet radiation, dehydration during low tide, and hypoxia during high tide. These cyclical stressors impose formidable challenges to foraging, locomotion, respiration, survival, and reproduction. Nevertheless, intertidal spiders of the genus *Desis* (family Desidae, see Figure 1A, Figure S1) have evolved a remarkable suite of physiological and behavioural adaptations that enable persistence under these extreme and fluctuating conditions. Unlike fully aquatic arthropods, *Desis* spiders exhibit activity patterns tightly entrained to tidal cycles. Individuals forage primarily during low tide, moving across exposed substrates and relying on effective terrestrial locomotion and well‐developed sensory systems. During high tide, they retreat into specialized silk structures that become fully submerged. Notably, these silk retreats function not only as protective shelters but also as air‐retaining ‘physical gills’, enabling sustained respiration throughout prolonged periods of submersion. Although partial ecological convergence is observed in the spray‐zone spider *Amaurobioides* (Araneae: Anyphaenidae), its habitat is only intermittently flooded and does not experience sustained submersion (Ceccarelli et al. 2016). Similarly, the jumping spider *Maratus marinus* possesses a suite of potential adaptations to marine‐influenced habitats, yet its maximum submergence duration is estimated to be approximately 1 h (Leggett et al. 2025). In contrast, true intertidal species such as *Desis jiaxiangi* experience regular immersion in seawater and can survive for more than 2 weeks within submerged silk retreats (Li et al. 2021; McQueen and McLay 1983). This extreme ecological specialization provides an unprecedented opportunity to explore how the molecular and genomic innovations that enables intertidal spiders to cross the boundary between land and sea.

Despite substantial advances in spider genomics—particularly in understanding the biomechanics of spider silk and the evolution of spidroin gene families (Babb et al. 2017)—genomic resources for spiders occupying marine or intertidal environments remain strikingly limited. To date, no chromosome‐scale genome has yet been assembled for a true intertidal spider. Consequently, the molecular mechanisms underlying key adaptive challenges in this environment—such as ionic homeostasis under saline conditions, oxygen management during prolonged submersion and silk specialization for underwater nesting—remain virtually unexplored.

Here, we present the first chromosome‐scale genome for the representative intertidal spider *D. jiaxiangi*, together with a reference genome of the water spider *Argyroneta aquatica* , integrated with comprehensive transcriptomic and proteomic analyses. Using this multi‐omics framework, we identify significant expansions in gene families associated with hormone biosynthesis (e.g., *farnesol dehydrogenase*) and DNA repair (e.g., *AlkB*). We further detect signatures of positive selection in genes involved in osmoregulation (e.g., *ATP1B*), sensory perception (odorant‐binding protein, *OBP*) and the rate‐limiting enzyme of glycolysis (*GCK*), as well as intensified selection on the mitochondrial regulator *ESRRB*. Furthermore, we uncover a novel GVGAKV motif in the spider *Sp* spidroin of *D. jiaxiangi*, which may potentially enhance silk hydrophobicity and facilitate underwater function. Notably, we observe amplification and diversification of hemocyanin genes, likely supporting oxygen transport and storage during extended submersion. Collectively, our genomic resources and analyses provide novel insights into the evolutionary innovations that enable spiders to thrive in the intertidal zone and shed light on broader genomic strategies underlying adaptation to extreme and transitional environments.

## 2 Materials and Methods

### 2.1 Genome and Transcriptome Sequencing

To assemble the genomes of *D. jiaxiangi* and *Argyroneta aquatica* , we collected adult female *D. jiaxiangi* specimens from the intertidal zone of Hainan Island, China, and *Argyroneta aquatica* specimens from Xilin Gol League, Inner Mongolia, China. To minimize microbial contamination, female spiders were dissected on ice to remove abdominal tissues containing intestinal microorganisms. Cephalothorax and leg tissues were immediately snap‐frozen in liquid nitrogen and used for genomic DNA (gDNA) extraction and whole‐genome sequencing.

We employed a combined sequencing approach using second‐ and third‐generation sequencing technologies. For both species, 500‐bp Illumina short‐insert libraries were prepared for second‐generation sequencing. For *D. jiaxiangi*, an additional 20‐kb PacBio long‐read library was prepared for third‐generation sequencing. Sequencing was performed on an Illumina HiSeq X Ten platform (paired‐end 150 bp, PE150; Illumina, USA) and a PacBio Sequel II system using single‐molecule real‐time (SMRT) sequencing. Raw sequencing reads were subjected to quality control to filter out adaptor sequences, low‐quality reads, and short fragments. After filtering, approximately 100 Gb of high‐quality Illumina reads (~46× coverage) and 101 Gb of PacBio long‐reads (~46× coverage) were retained for *D. jiaxiangi*, yielding a combined coverage of ~92× based on an estimated genome size of 2.18 Gb.

For transcriptome (RNA‐seq) analysis, three biological replicates were collected for each sample type. Female spider tissue samples included whole body, abdomen (containing all silk glands), book lungs, legs, venom glands and the complete silk gland complex under two experimental conditions: prolonged seawater submergence (> 24 h) and aerial exposure. Live spiders were dissected on ice, and tissues were immediately snap‐frozen in liquid nitrogen and stored at −80°C until use. Whole‐body samples from freshly collected spiders were also directly snap‐frozen in liquid nitrogen and stored at −80°C for RNA extraction.

Total RNAs were extracted using a TRIzol kit (Invitrogen, USA). Only RNA samples meeting quality criteria for concentration, purity, and integrity were used for library construction. RNA‐seq libraries were prepared following BGI standard protocols and sequenced on an Illumina HiSeq X Ten platform (PE150). Raw RNA‐seq reads were processed for quality control to remove adaptor sequences and low‐quality reads (Table S25).

To generate a high‐quality chromosome‐level reference genome for *A. aquatica* , we integrated publicly available raw Nanopore long‐read sequencing data (NCBI accession: SRR9994193) from Fan et al. (2025) with our newly generated Illumina short reads, Hi‐C data and RNA‐seq data (see Data Availability Statement).

### 2.2 Genome Survey and Assembly

We estimated genome size, heterozygosity and complexity of *D. jiaxiangi* using high‐quality Illumina short reads. A 17‐mer depth frequency distribution was generated, and K‐mer depth corresponding to the major peak was used to estimate genome size according to the formula: genome size = K‐mer number/K‐mer depth. The minor peak to the left of the main peak was used to infer genome heterozygosity, with higher peak height indicating greater heterozygosity, whereas peaks to the right of the main peak were used to assess genome duplication and overall genome complexity, with higher peak height indicating increased duplication and greater genome complexity.

PacBio long‐reads of *D. jiaxiangi* were assembled de novo using wtdbg2 v2.5 (Ruan and Li 2020). The resulting contigs were filtered and polished using NextPolish v1.3.1 (Hu et al. 2019), incorporating Illumina short reads to correct sequencing errors and improve assembly accuracy.

In contrast, Nanopore long reads of *A. aquatica* were assembled using NextDenovo (https://github.com/Nextomics/NextDenovo), which demonstrated superior performance for this dataset. The resulting contigs were subsequently polished with Illumina short reads using NextPolish v1.3.1 to correct sequencing errors.

The final assembly completeness and quality for both genomes were assessed using BUSCO v5.7.0 (Simão et al. 2015) with the arachnida_odb10 dataset.

### 2.3 Hi‐C Sequencing and Chromosome‐Level Genome Construction

To resolve long‐range chromatin interactions and construct chromosome‐level genome assemblies for *D. jiaxiangi* and *Argyroneta aquatica* genomes, we generated Hi‐C sequencing data. Cross‐linked gDNA was digested with MboI restriction endonuclease, and Hi‐C libraries were sequenced on the Illumina HiSeq platform using the PE150 sequencing strategy. Raw Hi‐C reads were processed using HiC‐Pro version 2.8.0_devel (Servant et al. 2015) for read alignment, filtering, and interaction matrix construction, yielding a set of valid chromatin interaction matrices.

Chromosome‐scale genome assemblies for *D. jiaxiangi* and *Argyroneta aquatica* were generated using the 3D‐DNA *de novo* genome assembly pipeline (version 180,922) (Dudchenko et al. 2017), which integrates Hi‐C interaction data from HiC‐Pro with PacBio long‐read assemblies. The resulting scaffolds were meticulously visualized and manually curated using Juicebox v1.11.08 (Durand et al. 2016) to identify and correct potential misassemblies. Unreliable joins were removed or adjusted, and the final chromosome‐level genome assemblies were generated following post‐review with 3D‐DNA.

### 2.4 Genome Annotation

Genome annotation comprised repeat identification and gene structure prediction. For repeat annotation, we established a species‐specific repetitive sequence database using RepeatModeler v2.0.1 (Flynn et al. 2020) and LTR_FINDER v1.07 (Xu and Wang 2007). RepeatMasker v4.1.2 (Tarailo‐Graovac and Chen 2009) was then used to identify and mask repetitive elements based on homology. The results were integrated and filtered to eliminate redundancy, generating a non‐redundant repeat annotation set.

Gene structure annotation was performed using a combination of ab initio, homology‐based, and transcriptome‐based approaches. For ab initio annotation, repetitive regions in the genome assembly were masked with N before gene prediction using Augustus v2.5 (Stanke and Waack 2003). A spider‐specific Augustus model was trained using annotated genomes of *Stegodyphus mimosarum* and *Trichonephila clavipes* .

For homology‐based prediction, annotated protein sequences from *S. mimosarum* , *T. clavipes* and *Parasteatoda tepidariorum* genomes were downloaded from NCBI and aligned to the target genomes using tblastn (Gertz et al. 2006) to identify putative gene loci. GeneWise v2.4.1 (Birney and Durbin 2000) was then used to refine exon‐intron structures and generate accurate gene models.

Transcriptome‐based prediction used RNA‐seq data from multiple tissues of intertidal and water spiders. After quality control with Trimmomatic v0.38167 (Bolger et al. 2014), reads were aligned to the assembled genome using HISAT2 v2.2.1 (Kim et al. 2019). Transcript structures were reconstructed using Stringtie v2.1.4 (Shumate et al. 2022).

Gene models from all three approaches were integrated using MAKER v2.31.8 (Holt and Yandell 2011), producing a unified, non‐redundant gene annotation. Functional annotation of predicted proteins was conducted by searching against Interpro (Blum et al. 2020), KEGG (https://www.kegg.jp/) and SwissProt (The UniProt Consortium 2016) databases.

Gene prediction and functional annotation for *A. aquatica* were performed using the same pipeline, incorporating its RNA‐seq data. Assembly quality and annotation completeness were evaluated using standard metrics and BUSCO assessment.

For comparative genomic analysis, chromosome‐level genomes of *D. jiaxiangi* (DEJI), *A. aquatica* (ARAQ), *Pardosa pseudoannulata* (PAPS) and *Oedothorax gibbosus* (OEGI) were performed using the MCscan pipeline (https://github.com/tanghaibao/jcvi/wiki/MCscan‐(Python‐version)). Synteny blocks were identified using default settings. The main steps included covariance comparison using ‘python3 ‐m jcvi.compara.catalog ortholog sp1 sp2 ‐‐no_strip_names’, followed by generating a simple file using ‘python3 ‐m jcvi.compara.synteny screen ‐‐minspan=30 ‐‐simple sp1.sp2.anchors sp1.sp2.anchors.new’ and ‘python3 ‐m jcvi.graphics.karyotype seqid layout’. Since the four spiders belong to different families, we adjusted the minimum block size to genes ‘‐‐minspan = 10’, to accommodate comparisons among species from different spider families.

### 2.5 Prediction of Demographic History

Demographic history was inferred using Pairwise Sequentially Markovian Coalescent (PSMC) model (Li and Durbin 2011), which reconstructs historical changes in effective population size based on the distribution of heterozygous sites across the diploid genome. PSMC estimates the time to the most recent common ancestor (TMRCA) along the genome using a Hidden Markov Model, allowing inference of population size fluctuations through time.

We applied PSMC to the *D. jiaxiangi* genome using parameters ‘g = 1, u = 5.4e‐09’, based on observed life cycle traits and a spider‐specific mutation rate (Mattila et al. 2012). Changes in effective population size were subsequently examined in relation to historical sea‐level fluctuations.

### 2.6 Phylogenomic Analysis and Divergence Time Estimation

We conducted a comprehensive comparative analysis of gene families within the subphylum Chelicerata, using the intertidal spider *D. jiaxiangi* as the reference species, incorporating 51 representative taxa (Tables S7 and S8). A total of 166 single‐copy orthologous genes were identified using BUSCO analysis, aligned with MUSCLE v3.8.31 (Edgar 2004), and trimmed with trimAl v1.4 using a heuristic automatic method (Capella‐Gutiérrez et al. 2009). Phylogenetic trees were reconstructed using the maximum likelihood (ML) method with IQ‐TREE v2.3.3 (Minh et al. 2020), which offers an ultrafast bootstrap (Hoang et al. 2017) algorithm and supports the Edge‐linked proportional partition model for assigning different substitution models to each sequence in the concatenated supermatrix. Substitution models were selected for each gene using the built‐in ModelFinder (Kalyaanamoorthy et al. 2017) under the Bayesian Information Criterion (‐MFP). For the concatenated supermatrix (338,666 bp), branch support was assessed using 1000 ultrafast bootstrap replicates with hill‐climbing nearest neighbour interchange (NNI) optimization (‐B 1000 ‐bnni ‐p FILE).

Divergence times were estimated using MCMCTREE in PAML v4.10.7 (Yang 2007) with approximate likelihood computation. Five fossil calibration points were applied (Tables S10 and S11). Each alignment was processed separately, and the General Time Reversible (GTR) substitution model (model = 7) was applied. Analyses were ran for 1000,000 steps, sampling every 10 steps, with the first 10,000 samples discarded as burn‐in (burnin = 10,000, sampfreq = 10, nsample = 100,000). Convergence was assessed using Tracer v1.7.2 (ESS > 200).

Ancestral habitat states of the marronoid spider clade were reconstructed using the PhyTools (http://www.phytools.org) R package.

### 2.7 Gene Family and Natural Selection Analysis

We analysed gene family evolution using the longest transcript per gene for each species. Protein sequences from 12 representative species were clustered into orthogroups and orthologs using OrthoFinder v2.5.5 (Emms and Kelly 2019). Single‐copy and multi‐copy orthologous genes, unique paralogous genes and unclassified genes for each species were identified. Single‐copy orthologs were aligned with MUSCLE v3.8.31 and concatenated into a single dataset for phylogenetic reconstruction using IQ‐TREE v2.3.3, with branch support evaluated via ultrafast bootstrap (1000 replications) and the Shimodaira‐Hasegawa approximate likelihood ratio test (1000 replications). The resulting phylogenetic tree was visualized with FigTree v1.4.4 (http://tree.bio.ed.ac.uk/software/figtree).

Positive selection and accelerated evolution analyses were performed using codeml in PAML v4.4. Branch models (one‐ratio vs. two‐ratio) were applied to detect rate shifts along the intertidal spider lineage (i.e., foreground branch), while branch‐site models (Test 2) were used to identify positive selected sites. The alternative Model A (allowing for sites with *ω* > 1 on the foreground branch) compared against the stringent Model A Null (ω fixed to 1). Significance was assessed using a Likelihood Ratio Test (LRT): 2Δℓ = 2 (lnL_alternative_ −lnL _null_), and compared to a Chi‐square distribution (df = 1). To control the false discovery rate, *p*‐values were corrected for multiple testing using the Benjamini‐Hochberg procedure. Positively selected genes (PSGs) were defined as those meeting either of the following stringent criteria: (1) adjusted *Q*‐value < 0.05; or (2) raw *p*‐value < 0.05 with high‐confidence positively selected sites (Bayes Empirical Bayes (BEB) posterior probability > 0.95). This approach minimizes false positives while retaining genes under strong, site‐restricted selection.

To capture different modes of adaptive evolution and complement PAML's site‐specific sensitivity, we used the HyPHY package (Pond et al. 2005). BUSTED (Murrell et al. 2015) was used to test for gene‐wide episodic selection, providing a robust assessment of overall evolutionary constraints, whereas PAML is optimized for detecting specific amino acid changes. RELAX (Wertheim et al. 2015) was used to distinguish between relaxation of purifying selection and intensification of positive selection. For both BUSTED and RELAX, *p*‐values were corrected for multiple testing using the Benjamini‐Hochberg FDR. Genes were considered under positive selection if they were significant in BUSTED (*Q* < 0.05) or PAML branch‐site model (*Q* < 0.05 or Raw *p* < 0.05 with BEB > 0.95 sites). For RELAX, significant genes were further classified into intensified (*K* > 1) or relaxed (*K* < 1) based on the selection intensity parameter (*K*).

Protein structures were predicted using AlphaFold3 (Abramson et al. 2024), visualized in PyMOL. Electrostatic potentials were calculated using APBS (Molecular Graphics System, Version 3.0 Schrödinger LLC). Thermodynamic stability changes (ΔΔG) upon mutation were predicted with the DUET web server (https://biosig.lab.uq.edu.au/duet/). DUET integrates graph‐based signatures (mCSM) and statistical potential energy (SDM) for consensus predictions. Negative ΔΔG indicates destabilization (increased flexibility/solvent exposure), whereas positive ΔΔG indicates stabilization.

Gene family expansion and contraction were analysed using CAFE v5.0 (Mendes et al. 2020). Gene families with *p*‐value < 0.05 were considered significantly expanded or contracted. To explore the above significant genes in *D. jiaxiangi*, we extracted protein sequences, compared them to the NR database and performed GO and KEGG enrichment analyses using the ‘enrichGO’ function in clusterProfiler v4.0 (Xu et al. 2024).

### 2.8 Transcriptome Analyses

RNA‐seq reads were assembled, annotated and analysed. Using the assembled and annotated genome sequences and protein coding sequences described previously, transcriptome data were aligned to the reference to the genome using STAR v2.7 (Dobin et al. 2012). Gene expression levels were quantified using featureCounts (Liao et al. 2013) from subread v2.0.6. Differential expression analyses of different tissue samples were conducted using DESeq2 v1.26 (Love et al. 2014). Genes with a *padj* < 0.05 and a fold change > 2, after multiple testing correction, were considered differentially expressed. Functional enrichment analyses were performed using GO and KEGG databases.

### 2.9 Proteomic Analyses

Adult *D. jiaxiangi* spiders (*n* = 49) were collected from Hainan Island during low tide in September 2019 and maintained under laboratory conditions simulating the intertidal environment (salinity 35‰). Due to limited nest production, silk samples were obtained through repeated extractions. Six individuals (three females and three males) were selected and sampled in triplicate (female: Fx; male: Mx) designed as 205Fx, 226Fx, 221Fx, 250 Mx, 241 Mx, and 203 Mx, respectively. All silk samples were cryopreserved at −20°C prior to protein extraction.

For protein extraction, silk samples were transferred to 2 mL centrifuge tubes containing steel beads and Lysis Buffer 3 (including SDS, EDTA, and a protease inhibitor cocktail) supplemented with 10 mM dithiothreitol (DTT). Samples were homogenized using a tissue grinder (60 Hz for 2 min) to mechanically disrupt silk fibres, followed by incubation at 56°C for 1 h to ensure complete solubilization and reduction of disulfide bonds. Proteins were subsequently alkylated with 55 mM iodoacetamide (IAM) in the dark for 45 min. To remove interfering substances, proteins were precipitated with cold acetone (1:5 v/v) at −20°C for 30 min. The resulting protein pellets were air‐dried and resuspended in an SDS‐free buffer. Protein concentrations were quantified using the Bradford assay. Proteins were digested with trypsin at an enzyme‐to‐substrate ratio of 1:20 at 37°C for 4 h. Peptide labeling and mass spectrometry analyses were conducted on a BGI mass spectrometry platform.

Raw mass spectrometry data were converted to mgf format files and searched against a custom arachnid protein database using Mascot v2.3.02. Custom database comprised known spidorin sequences retrieved from NCBI, genome‐predicted protein sequences from *D. jiaxiangi*, and transcriptome‐annotated gene‐protein sequences. Quality control was performed to ensure data validity, and final protein identifications were accepted based on the following criteria: fragment mass tolerance of 0.02 Da, peptide mass tolerance 10 ppm and the presence of at least one specific peptide.

iTRAQ quantitative proteomic analysis was conducted using IQuant software (BGI), with 1% FDR filtering at the profile or peptide level (PSM‐level FDR ≤ 0.01). Proteins were assembled from identified peptides following the principle of parsimony and further filtered using a picked protein FDR strategy to maintain a protein‐level FDR ≤ 0.01. Differentially abundant proteins of interest were identified based on quantitative outputs from IQuant. Functional annotation of identified proteins was performed by aligning against the NCBI NR, GO and KEGG databases.

### 2.10 Spidroin, Hemocyanin and Chemoreceptor Gene Family Analyses

We conducted an in‐depth analysis of the spidroin, hemocyanin and chemoreceptor gene families using both the assembled chromosome‐level genome assemblies and transcriptome data. To identify spidroin genes in *D. jiaxiangi* and *A. aquatica* , translated protein sequences from both genomes were queried against the NCBI non‐redundant (NR) protein database (April 2023 release). Candidate spidroins were validated based on the presence of characteristic repetitive motifs, phylogenetic relationships with known spidroins from *Pardosa pseudoannulata* , and manual curation.

To examine the evolutionary relationships of hemocyanin genes across arthropods, hemocyanin protein sequences from representative species were retrieved from the NCBI database (Table S24). For annotation of major chemoreceptor gene families in *D. jiaxiangi*, we used the BITACORA pipeline (Vizueta et al. 2020), which integrates homology‐based searches with iterative annotation refinement. Multiple sequence alignments were performed using Muscle v5 (Edgar 2022). Phylogenetic trees were reconstructed using the maximum likelihood method implemented in IQ‐TREE v2.1.3 (Minh et al. 2020) and visualized with FigTree v1.4.4.

### 2.11 Hydrophobicity Analyses

Water contact angle measurements were conducted using an Attension Theta goniometer, with data processing in OneAttension v1.8 software (Nanoscience Instruments, USA). Spider webs were collected within 3 days of production and mounted flat on glass coverslips. Each experimental condition was tested in triplicates, with ten 10‐μL water droplets applied to different locations on each sample.

To assess whether the hydrophobicity of *D. jiaxiangi* silk retreats represents a species‐specific adaptation or a generic feature of spider silk lipids, we measured contact angles (CA) of webs and egg sacs from terrestrial species ( *Allagelena difficilis* , *Parasteatoda tepidariorum* , and *Pardosa pseudoannulata* ) and the water spider ( *Argyroneta aquatica* ). Statistical analyses were performed using OriginLab (OriginLab, USA).

For ultrastructural examination, silk samples were sputter‐coated with a 10‐nm platinum layer and imaged using a field‐emission scanning electron microscope (SEI mode, 10–15 keV, JSM‐7600F, JEOL).

Motif consensus sequences were identified using the MEME suite with a minimum motif width of 3. Detected motifs included GVGAK (46 sites; *e*‐value = 5.3e‐74), ERLLLCKKYC (10 sites; *e*‐value = 7.7e‐15), and YDJLINADC (19 sites; *e*‐value = 1.5e‐9). Secondary structures and solvent accessibility were predicted using the RaptorX algorithm (Wang et al. 2016). Three‐dimensional protein structures were predicted using AlphaFold2 implemented in ColabFold (Jumper et al. 2021), modelling 4‐mer of the 10× GVGAKV motif with a recycling limit of 20, yielding an overall pLDDT score of 85.

## 3 Results

### 3.1 A Chromosome‐Scale Genome Reveals the Genomic Architecture of *D. jiaxiangi*

To understand the genetic basis of intertidal adaptations, we assembled a high‐quality, chromosome‐scale genome assembly for *D. jiaxiangi*. While the estimated genome size is approximately 2.18 Gb (Figure S2, Table S1), the final assembled genome spans 1.97 Gb, with a contig N50 exceeding 1 Mb (Table S2) and 97.5% BUSCO completeness (Table S3). HiC data (Figure S3) identified 22 chromosomes in the haplotypic assembly (Figure 1B), ranging from 56 Mb to 126 Mb in length and achieving a scaffold N50 of 90.3 Mb (Table S4). Repeat annotation revealed that repetitive sequences comprise approximately 48.08% of the genome (Table S5). Gene structure annotation identified 24,931 protein‐coding genes, with an average coding sequence (CDS) length of 1144 bp (Table S6).

In comparison, the water spider *A. aquatica* was found to possess a haploid chromosome number of 11. Its genome assembly is relatively compact, totaling approximately 0.79 Gb, with a contig N50 of 14 Mb and 97.5% BUSCO completeness (Tables S2 and S3). Repetitive sequences were substantially reduced in this species, accounting for only ~333 Mb of the assembly (Table S12), markedly lower than in *D. jiaxiangi* and other spider genomes analyzed here.

To investigate chromosome evolution within the retrolateral tibial apophysis (RTA) clade, we conducted synteny analyses among *D. jiaxiangi* (Desidae), *A. aquatica* (Argyronetidae) and *Pardosa pseudoannulata* (Lycosidae) (Yu et al. 2024), using *Oedothorax gibbosus* (Linyphiidae) (Hendrickx et al. 2021) as an outgroup due to the availability of chromosome‐level genomic data (Figure 1B). These comparisons revealed extensive conservation of syntenic blocks alongside lineage‐specific chromosomal rearrangements. Notably, we detected a two‐to‐one chromosome fusion or fission event between *D. jiaxiangi* and *A. aquatica* during chromosome evolution, consistent with their shared evolutionary history within the RTA clade.

### 3.2 Demographic History Mirrors Pleistocene Sea‐Level Fluctuations

To assess how past environmental changes have shaped the evolutionary trajectory of *D. jiaxiangi*, we reconstructed its demographic history over the past one million years using coalescent‐based modelling. The inferred effective population size fluctuated substantially between 1 Mya and 10 Kya, broadly in line with Pleistocene sea‐level oscillations (Figure 1C). Population expansion occurred during glacial periods characterized by lower sea levels, when intertidal habitats would have been more extensive.

This demographic pattern closely parallels that reported for the intertidal amphibious mudskipper *Boleophthalmus pectinirostris* (You et al. 2014), suggesting that the expansion of intertidal zones during glacial maxima may have provided recurrent ecological opportunities for intertidal and amphibious organisms. In contrast, the effective population size of *D. jiaxiangi* has been declining since 100 Kya (Figure 1C), potentially reflecting habitat contraction associated with post‐glacial sea‐level rise.

### 3.3 Phylogenomics Clarify Evolutionary Origins

To place *D. jiaxiangi* in a broader evolutionary framework, we reconstructed a phylogeny of 49 spider species with available genomes exhibiting BUSCO completeness exceeding 75% (Figure 1D, Tables S7 and S8). A total of 166 single‐copy orthologous genes were identified using BUSCO‐based gene prediction (Table S9). The resulting tree topology is largely congruent with previous phylogenetic studies (Fernandez et al. 2018; Wheeler et al. 2016), with strong support for most nodes (100 bootstrap). However, one notable exception involves the placement of Eresidae. Contrary to its traditional assignment with the RTA clade, Eresidae was recovered as a sister group to Araneoidea, albeit with relatively weak bootstrap support (71%). This incongruence likely reflects limited taxon sampling and genomic representation for this family, underscoring the need for broader phylogenomic coverage.

Notably, *D. jiaxiangi* clusters most closely with the freshwater water spider *A. aquatica* , with an estimated divergence time of approximately 107 Mya (Figure 1D, Tables S10 and S11), coinciding with the Cretaceous Terrestrial Revolution (Lloyd et al. 2008). This estimate is consistent with previous mitogenome‐based analyses (Li et al. 2021). Ancestral character reconstruction further indicates that extant aquatic lineages within the marronoid spider clade originated independently from a terrestrial ancestor, with one lineage transitioning to freshwater habitats and another to marine intertidal environments (Figure 1D).

Comparative analyses of genome architecture revealed pronounced variation in genome size and repeat content across spider lineages. In particular, RTA spiders exhibit substantially greater genome size variability and higher proportions of repetitive elements compared with Araneoidea (Figure 1D, Table S12).

### 3.4 Expansion of Gene Families Associated With Hormone Biosynthesis and DNA Repair

To uncover the genomic basis of intertidal adaptation, we examined gene family evolution across annotated spider genomes (Figure S4A). In *D. jiaxiangi*, we identified 763 significantly expanded and 1245 contracted gene families (DEJI in Figure S4A).

KEGG and GO enrichment analyses of the expanded gene families revealed functional categories with clear relevance to environmental stress tolerance. KEGG pathways enrichment (Figure S4B, Table S13) highlighted significant expansion in pathways tied to insect hormone biosynthesis (ko00981), which is implicated in growth and developmental regulation; DNA repair and recombination proteins (ko03400), crucial for maintaining genomic stability; lysine degradation (ko00310), involved in energy metabolism and stress response (Battur et al. 2009); cell adhesion molecules (ko04515), essential for tissue integrity under mechanical and osmotic stress; p53 signalling pathway (ko04115), associated with apoptosis and DNA repair; and glutathione metabolism (ko00480), which plays a central role in detoxification and oxidative stress defence (Raj Rai et al. 2021).

GO enrichment analysis (Figure S4C, Table S14) reinforced these findings, revealing significant overrepresentation of biological processes including juvenile hormone and sesquiterpenoid metabolism (GO:0006716, GO:0016106), oxidative demethylation (GO:0070989), detection of mechanical stimuli (GO:0050976) and mitochondrial DNA replication (GO:0006264).

Notably expanded gene families include those encoding key enzymes involved in hormone synthesis (e.g., *farnesol dehydrogenase*, Figure S4D), DNA damage repair (e.g., *AlkB* and *RRM2*, Figure S4E,F), and cellular energy production. Collectively, these expansions likely contribute to enhanced stress tolerance and physiological resilience in the highly dynamic and physiochemically challenging intertidal environment.

### 3.5 Molecular Evolution of Genes Involved in Osmoregulation and Sensory Responses

To identify molecular signatures of adaptive evolution in intertidal spiders, we applied complementary codon‐based approaches implemented in PAML and HyPHY to detect positively selected genes (PSGs), rapidly evolving genes (REGs) and genes under intensified or relaxed selection along the intertidal spider lineage. By integrating the site‐specific sensitivity of PAML with the gene‐wide robustness of HyPHY (BUSTED and RELAX), we defined a comprehensive ‘adaptive evolution gene set’ (Figure S5A,B, Table S15). We then conducted functional enrichment analyses using GO (Figure S5C, Table S16) and KEGG (Figure S6, Table S17).

GO enrichment revealed a significant overrepresentation of terms associated with epithelial development, including ‘epithelial tube morphogenesis’ (GO:0060562, *p* = 5.26E‐05) and ‘branching morphogenesis’ (GO:0048754). Several key developmental regulators, including members of the SOX transcription factor family and MAGUK proteins involved in tight junctions, showed accelerated evolutionary rates. Consistently, KEGG pathways critical for epithelial integrity, such as ‘Gap junction’ (map04540) and ‘Adherens junction’ (map04520), were significantly enriched among rapidly evolving gene clusters.

Beyond epithelial and respiratory structures, we detected genomic signals indicative of exoskeletal reinforcement. GO terms such as ‘regulation of ossification’ (GO:0030278) and ‘cartilage development’—used here as functional proxies for chitinous sclerotization in arthropods—showed signatures of intensified selection (RELAX intensification). This pattern was accompanied by evidence of positive selection in the Hedgehog signalling pathway (map04340), a key regulator of cuticle patterning and thickness. In contrast, sensory systems showed a mosaic pattern of evolution: mechanosensory genes remained largely conserved, whereas several visual pathways (e.g., ‘Serotonergic synapse’, ‘Phototransduction’) exhibited signals of relaxed selection (Relaxation), consistent with the cryptozoic, cavity‐dwelling lifestyle of *Desis*.

**FIGURE 2:**
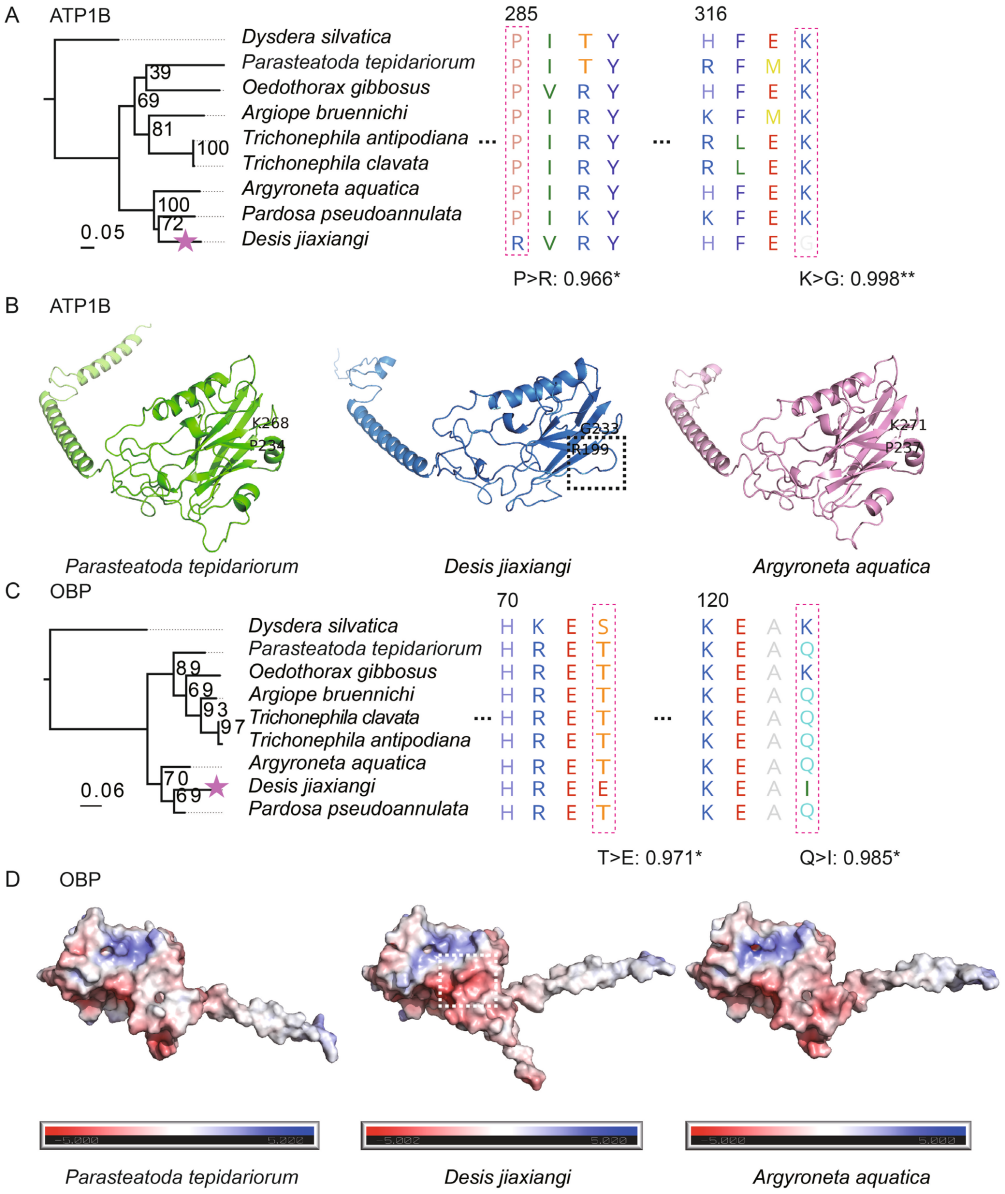
Phylogeny, positively selected sites and protein structures of ATP1B and OBP in three representative spider species. (A) Phylogeny and sequence alignment of ATP1B, with positively selected sites indicated. (B) Predicted protein structures of ATP1B; black dashed boxes highlight regions exhibiting structural variation. (C) Phylogeny and sequence alignment of OBP, with positively selected sites indicated. (D) Surface representation of the OBP protein showing electrostatic potential calculated using APBS; white dashed boxes denote altered charge distribution. Red indicates negative charge, and blue indicates positive charge.

To further characterize key genes underlying these adaptive signatures, we examined specific PSGs and REGs in detail. Sodium/potassium‐transporting ATPase subunit beta (*ATP1B*), which is involved in ion transport and osmoregulation, displayed two sites under strong positive selection (P: 0.966*; K: 0.998**; see Figure 2A). These substitutions suggest potential functional modification of ion transport dynamics relevant to saline environments. Structural predictions generated using AlphaFold3 (Abramson et al. 2024) for three spider species revealed lineage‐specific differences in helix (P) and loop structures (R) among *Pardosa pseudoannulata* (PATE), *Argyroneta aquatica* (ARAQ) and *D. jiaxiangi* (DEJI), particularly in regions implicated in protein flexibility (Figure 2B).

The odorant‐binding protein (*OBP*) gene also exhibited evidence of positive selection, with two significantly sites showing high posterior probabilities (E: 0.971*; I: 0.985*; Figure 2C). OBPs play a central role in chemosensory systems by binding and transporting hydrophobic odour molecules to olfactory receptors (Brito et al. 2016). Structural modelling indicated that the T73E substitution introduces a negatively charged residue, increasing surface polarity and local flexibility, and was predicted to be destabilizing (ΔΔG DUET = −0.495 kcal/mol). Conversely, the Q123I substitution replaces a polar residue with hydrophobic one, strengthening the protein core and was predicted to be stabilizing (ΔΔG DUET = +0.76 kcal/mol), potentially compensating for destabilizing effects elsewhere in the molecule (Figure 2C,D).

The gene *RHOGDI*, involved in cytoskeletal regulation and water reabsorption processes, contained one site under significant positive selection (BEB > 95%; Figure S7A,B). The observed substitution from Leucine (L) to Threonine (T) introduces a polar residue that disrupts local hydrophobic interactions, although no significant structural changes were observed. Finally, *CAPON*, a gene implicated in circadian entrainment, harboured two positively selected sites (BEB > 95%; Figure S7C,D). These substitutions, including a change from Glutamine (Q) to Alanine (A) and from Lysine (K) to Aspartic Acid (D), are predicted to alter local charge balance and protein–protein interaction potential, potentially affecting subcellular localization or signalling dynamics.

### 3.6 Gene Expression Supports Functional Adaptation

**FIGURE 3:**
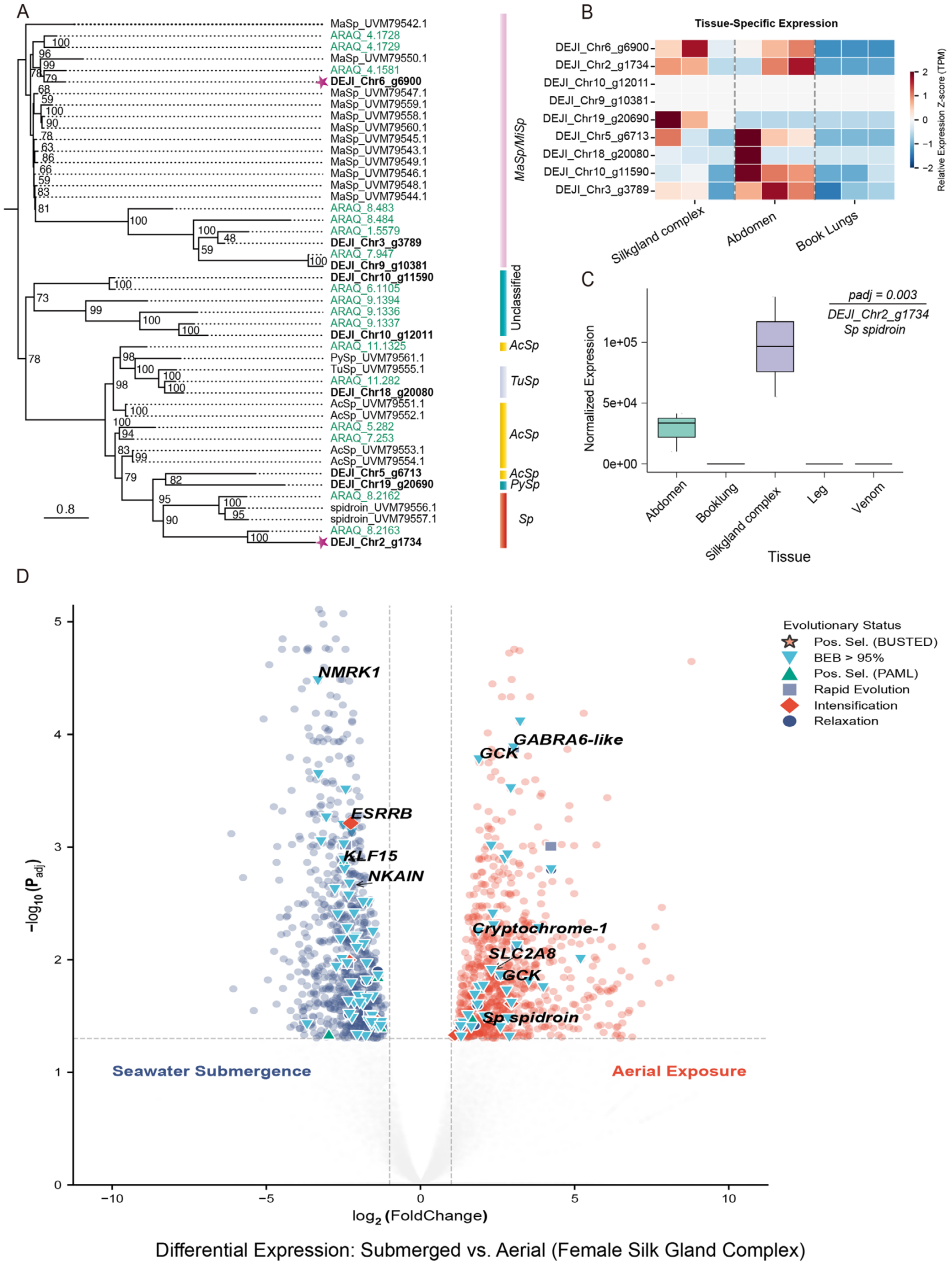
Phylogenetic relationships, expression patterns of spidroins and differential gene expression in *Desis jiaxiangi*. (A) ML phylogenetic tree constructed from full‐length spidroin sequences. Stars indicate proteomic evidence. *Desis jiaxiangi* (DEJI) IDs are shown in bold black; spidroins from *Argyroneta aquatica* (ARAQ) are shown in green, and those from *Pardosa pseudoannulata* (PAPS) are shown in black. (B) Heatmap showing relative expression levels (*z* score) of all DEJI spidroin genes across different tissues, as determined by bulk RNA‐seq. (C) Boxplot of DEJI *Sp spidroin* gene expression across tissues. Note that abdominal tissue includes the whole silk gland complex. (D) Differential gene expression analysis of the female silk gland complex under seawater submergence versus aerial exposure.

To link genomic signatures of adaptations with physiological function, we performed bulk RNA‐seq across multiple tissues (Figure 3, Figure S8) and mapped the resulting transcriptome profiles to the *D. jiaxiangi* reference genome. Analysis of the top 50 highly expressed genes in each tissue revealed consistently high expression of *hemocyanin* genes in the abdomen and venom gland tissues, highlighting their crucial role in oxygen transport.

To further assess the functional relevance of candidate adaptive genes, we performed a comparative transcriptomic analysis of the whole silk gland complex under two ecologically relevant environmental conditions (Figure 3, Table S18): seawater submergence (high tide) and aerial exposure (low tide). Differential expression analysis was performed using DESeq2, revealing distinct expression profiles associated with contrasting environmental demands.

During high tide, submergence was associated with transcriptional changes consistent with metabolic and physiological adjustment to hypoxic and osmotic stress. The transcription factor *KLF15* (Log2FC = −2.49), a master regulator of metabolic depression and amino acid gluconeogenesis (Jeyaraj et al. 2012), and *NMRK1* (Log2FC = −3.32), critical for NAD^+^ salvage under energetic stress, were differentially expressed. In parallel, the mitochondrial regulator *ESRRB* showed elevated expression during submergence (Log2FC = −2.26), potentially contributing to mitochondrial stability under low‐oxygen conditions. Notably, *NKAIN* (Na^+^/K^+^‐ATPase Regulator) was highly expressed in silk glands, suggesting a role in maintaining glandular ionic balance under extreme salinity fluctuations.

In contrast, aerial exposure triggered a pronounced transcriptional response indicative of increased metabolic and sensory activity. Multiple cytochrome P450 genes (e.g., *CYP4c3*, Log2FC > 2.8) and a *GABRA6‐like* gene (Log2FC = +3.02) were strongly upregulated, consistent with enhanced detoxification capacity and modulation of sensory signalling (Gingl et al. 2004). The gene encoding glucokinase (*GCK*), the rate‐limiting enzyme of glycolysis (Lin and Xu 2016), was both under positive selection (BEB support, *p* < 0.05) and significantly upregulated during air exposure (Log2FC = 1.89 and 2.59). We also detected strong upregulation of the Facilitated trehalose transporter *Tret1* (*SLC2A8*; Log2FC = 2.30). As trehalose is the primary hemolymph sugar supporting burst locomotion in arthropods (Kikawada et al. 2007), this pattern suggests rapid mobilization of energy reserve following emersion. Additionally, increased expression of *Cryptochrome‐1* (Log2FC = 1.73) indicates that tidal cycles may influence circadian entrainment. The *Sp spidroin* gene was significantly upregulated under aerial conditions, consistent with increased silk production demands during nest construction. Together, these expression patterns reveal rapid and coordinated transcriptional responses in silk glands that align with alternating osmotic, metabolic and mechanical challenges in the intertidal environment.

### 3.7 Silk Proteome Enriched With Hydrophobic Sp Spidroin and Hemocyanins

Given the crucial role of silk in intertidal spiders' adaptation, we conducted proteomic analyses to characterize the composition of *D. jiaxiangi* silk nests. Mass spectrometry (MS) identified 570,826 secondary spectra (Tables S19 and S20) and 219 proteins under a stringent 1% false discovery rate (FDR) criterion (Tables S21 and S22). Among these identified proteins were major ampullate spidroins (MaSp) and the Sp spidroin (Sp). Interestingly, we also detected eight hemocyanin subunit g sequences and 13 antimicrobial peptide (AMP) precursors (Table S23), suggesting additional roles for silk in oxygen storage and antimicrobial defense.

Genome annotation of *D. jiaxiangi* (DEJI) revealed five types of spidroin genes: *MaSp*, *AcSp*, *TuSp*, *PySp* and Sp. We combined these sequence data with spidroins from the freshwater water spider ( *Argyroneta aquatica* , ARAQ) and the semi‐aquatic pond wolf spider ( *Pardosa pseudoannulata* , PAPS) to reconduct a comparative phylogeny (Figure 3A). The resulting topology revealed that *Sp spidroin* from all three species clustered into a single clade (Figure 3A). Expression profiling confirmed that all recovered spidroin genes were transcriptionally active in silk gland‐related tissues, with *Sp spidroin* exhibiting significant overexpression in the silk gland complex (Figure 3B,C).

Comparative sequence analysis of Sp spidroin across the three RTA spiders revealed a high abundance of hydrophobic glycine‐valine (GV) motifs. Specifically, the GV motif occurred 74 times in *D. jiaxiangi* (DEJI_Chr2_g1734, see simplified gene ID in Table S26), 71 times in *A. aquatica* (ARAQ_8.2163) and 56 times in *P. pseudoannulata* (spidroin_UVM79557.1). Remarkably, *D. jiaxiangi* Sp spidroin contains a unique GVGAKV motif repeated 51 times, accounting for over 23% of the sequence, whereas this motif is entirely absent (0 repeats) in *A. aquatica* , which retains only the canonical GV motif (Correa‐Garhwal et al. 2019). Together, these results suggest that spiders occupying intertidal and aquatic habitats possess a higher density of hydrophobic GV‐based motifs in Sp spidroin compared with more terrestrial relatives, consistent with functional divergence of silk properties across ecological transitions.

### 3.8 Silk Web Hydrophobicity Is Facilitated by Sequence and Structure

**FIGURE 4:**
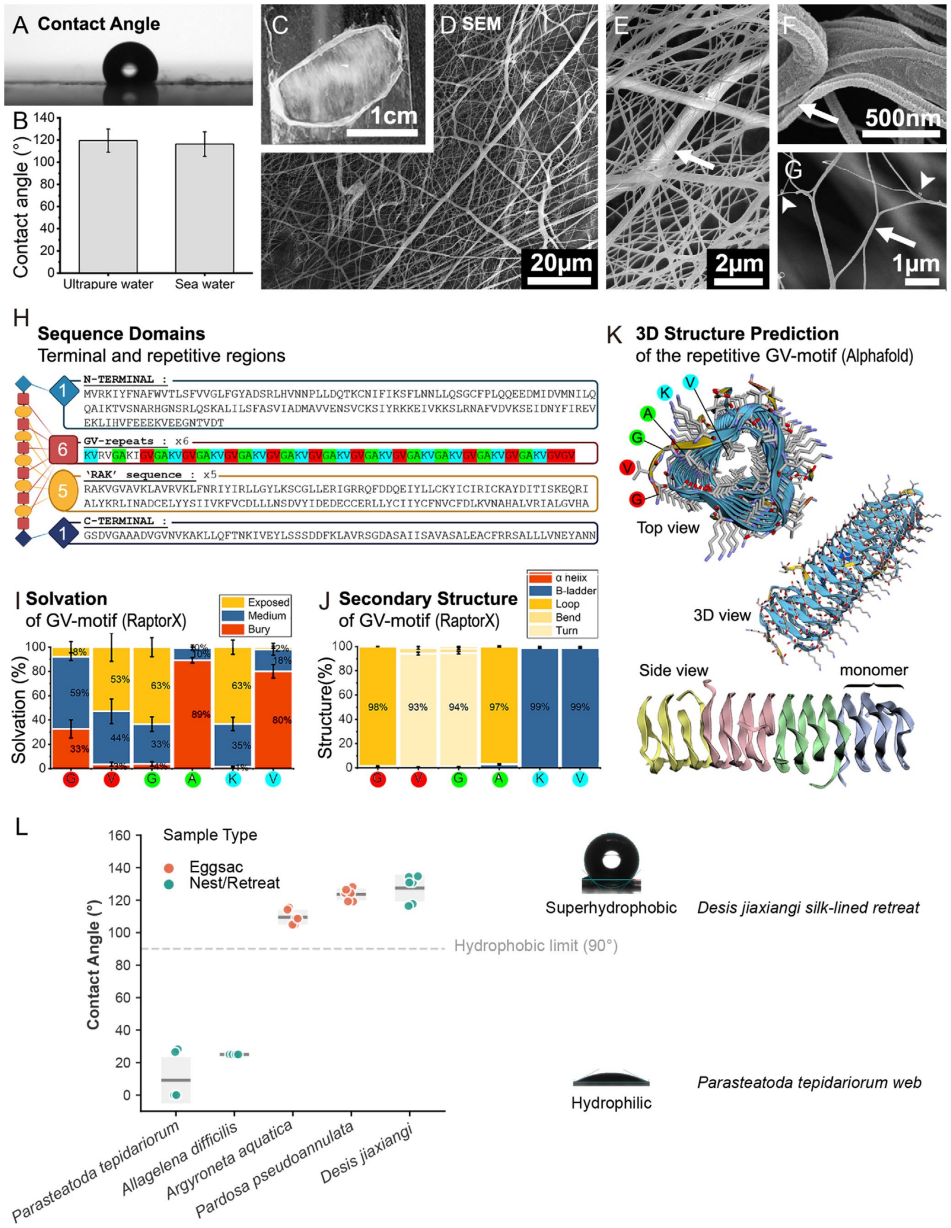
Hydrophobicity and structural characterization of the *D. jiaxiangi* silk‐lined retreat. (A) Representative contact‐angle images and (B) quantitative measurements using ultrapure water and seawater. (C) Photograph of the silk‐lined retreat deposited on a glass coverslip. (D–G) SEM imaging of silk fibres from the retreat. Arrows indicate interconnected fibres, and arrowheads highlight knots and curled fibre structures. (H) Schematic of functional domains in the protein sequence, showing N‐ and C‐termini with alternating GV‐rich and ‘RAK’ domains; GVGAKV motifs are highlighted. (I, J) Predicted solvation and secondary structure properties, respectively, generated using RaptorX and averaged across multiple GVGAKV motifs. (K) Predicted A 4‐mer 3D structure from the 10× GVGAKV repeats motif monomer, modelled using AlphaFold2. (L) Contact‐angle measurements of nests or egg sacs from five spider species. Terrestrial species include *Parasteatoda tepidariorum* , *Allagelena difficilis* and *Pardosa pseudoannulata* ; aquatic species include *Argyroneta aquatica* and *Desis jiaxiangi*.

We next examined the hydrophobic properties of *D. jiaxiangi* silk retreats. Contact angle measurements revealed mean wetting angles of 119° (12 samples) for ultrapure water and 116° (7 samples) for seawater, with a standard deviation of ±10° (Figure 4A,B), reflecting the inherent heterogeneity of natural silk samples. Both values indicate strong hydrophobicity.

Optical and SEM images revealed that the retreat web is composed of densely interwoven silk fibres of varying diameters, forming a mesh with inter‐fibre gaps predominantly smaller than 2 μm (Figure 4C–G). This fine‐scale architecture is critical for intertidal survival. From a physical perspective, the combination of narrow pore size (< 2 μm) and hydrophobic fibre surfaces promotes a Cassie‐Baxter wetting state, in which air is retained within the textured mesh. As a result, the silk retreat functions as a waterproof air chamber: during high tide, the hydrophobic silk network traps a stable air layer, resisting hydrostatic pressure and preventing the spider from seawater penetration, thereby enabling prolonged submersion without drowning until the tide recedes.

Comparative measurements showed that capture webs of the terrestrial house spider *Parasteatoda tepidariorum* (Theridiidae) and the funnel weaver *Allagelena difficilis* (Agelenidae) were hydrophilic (contact angle < 90°, Figure 4L). In contrast, the egg sacs of the pond wolf spider *Pardosa pseudoannulata* (Lycosidae) exhibited strong hydrophobicity (contact angle > 120°). Notably, the wetting properties of the intertidal spider *D. jiaxiangi* silk retreats closely resemble those of terrestrial spider egg sacs rather than capture webs (Figure 4L).

At the molecular level, the Sp spidroin consists of alternating, GV‐rich domains and conserved ‘RAK’ motifs, flanked by N‐terminus (NT) and C‐terminus (CT) domains (Figure 4H). Given the established association between GV motifs and hydrophobicity (Correa‐Garhwal et al. 2019), we analysed motif distribution using the MEME algorithm (Bailey and Elkan 1994), and identified six regions containing low‐complexity repeats of 10× GVGAKV. These regions corresponded to reduced alpha‐helix propensity in secondary structure predictions generated by RaptorX algorithm (Figure 4J, Figure S9C,G,H).

To explore structure implications, we used AlphaFold2 (Jumper et al. 2021) to predict the 3D structure of a GV‐rich tetramer. The resulting model revealed an amyloid‐like structure (Kenney et al. 2002) (Figure 4K) characterized by exposed aliphatic Valines residues and periphery Lysines residues, a configuration consistent with enhanced surface hydrophobicity. Solvent accessibility analysis using RaptorX (Wang et al. 2016) further supported this interpretation (Figure 4I,S9E).

BLAST search of the N‐terminus (NT) domain revealed strong sequence homology to *Stegodyphus dumicola* , *Araneus diadematus* and *Octonoba sybotides* , consistent with a conserved role in spidroin multimerization. Structural modelling of NT‐CT domain interactions further supported this function (Figure S9A). Additionally, the five highly similar ‘RAK’ domains were predicted to adopt alpha‐helix secondary structures (Figure S9B,D,F,H). These helices are likely to form an interconnecting network within the silk fibre, providing elasticity through reversible deformation under mechanical strain, thereby contributing to the mechanical robustness of the retreat silk (Gosline et al. 1999).

### 3.9 Hemocyanin Expansion Suggests Adaptation to Hypoxic Environments

The unexpected detection of hemocyanin in the silk proteome of *D. jiaxiangi* prompted a comparative genomic analysis across intertidal (*D. jiaxiangi*), aquatic (water spider) and terrestrial arthropods (Table S24).

**FIGURE 5:**
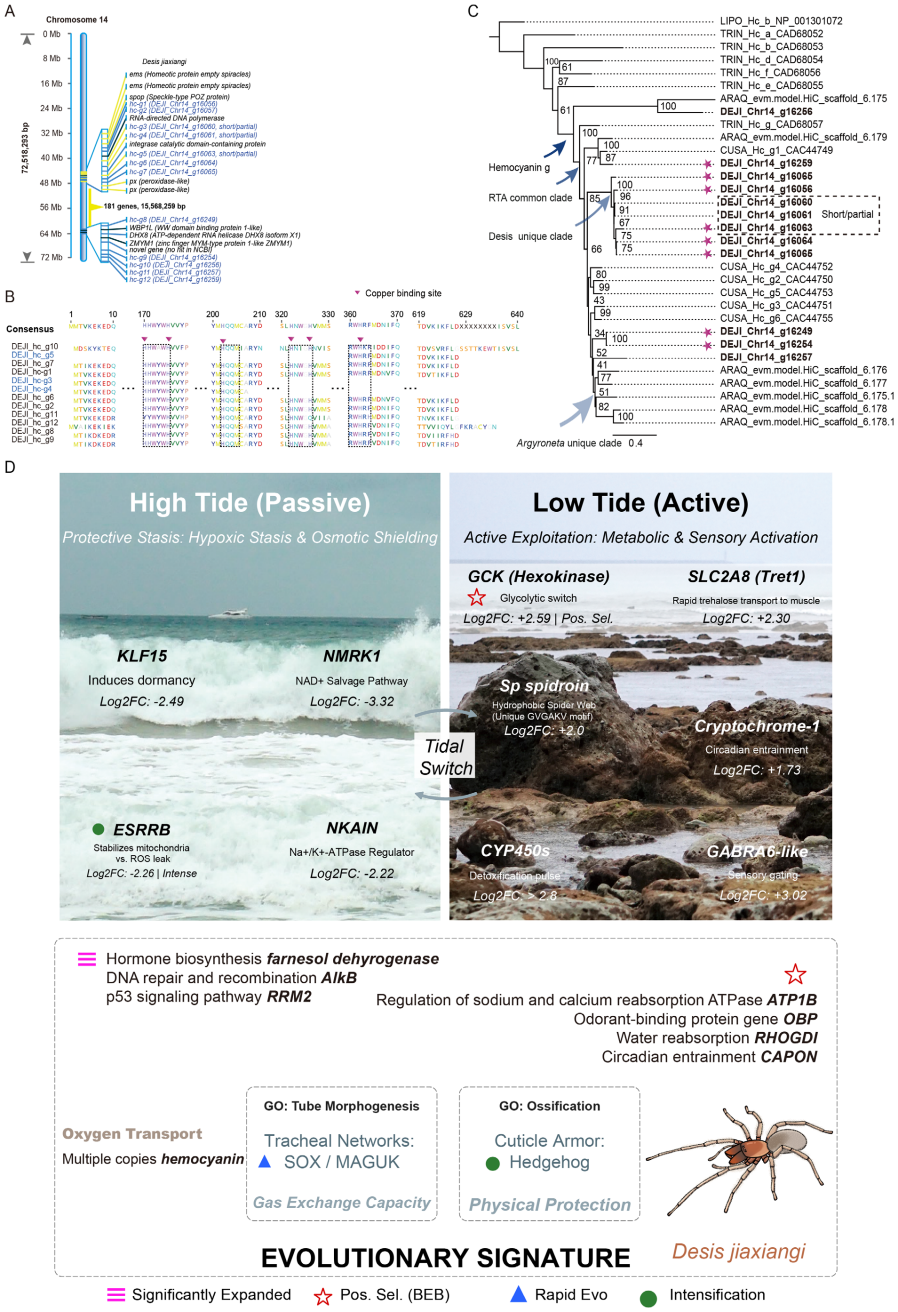
Hemocyanins in *D. jiaxiangi* and a graphical summary of this study. (A) Genomic organization of hemocyanin (*hc*) genes and their adjacent genes on the Chr 14 of *D. jiaxiangi*. (B) Sequence alignment of all hemocyanins in *D. jiaxiangi*; truncated or partial hemocyanins are highlighted in blue. (C) Phylogenetic relationships among arthropod hemocyanins. Stars indicate proteomic evidence, and *D. jiaxiangi* (DEJI) hemocyanin IDs are shwon in bold black. Species abbreviations: LIPO, *Limulus polyphemus* ; TRIN, *Trichonephila inaurata madagascariensis* ; ARAQ, *Argyroneta aquatica* ; CUSA, *Cupiennius salei* . (D) Summary of genes exhibiting lineage‐specific evolutionary signatures in *D. jiaxiangi* and their inferred adaptive functions. The digital illustration of the intertidal spider was created by Bernetta Kwek (2024, Singapore).

Genomic localization analysis revealed that all hemocyanin genes in *D. jiaxiangi* are clustered on chromosome 14, where they form two distinct gene clusters separated by a peroxidase gene (*px*) (Figure 5A). The intervening *px* gene, positioned adjacent to the upper hemocyanin gene (DEJI_Chr14_g16056), may contribute to aerobic respiration by processing reactive oxygen byproducts, although its precise functional relationship to hemocyanin remains to be determined.

Of the 12 hemocyanin genes identified in *D. jiaxiangi*, nine retain six conserved histidine residues required for copper binding at the active site ions, consistent with canonical oxygen‐transport function. In contrast, three genes are truncated: DEJI_Chr14_g16060 and DEJI_Chr14_g16061 retain only three histidine residues, whereas DEJI_Chr14_g16063 retains a single histidine residue. This structural variation suggests functional divergence or partial loss of oxygen‐binding capacity among hemocyanin paralogs (Figure 5B).

Phylogenetic analysis revealed that all examined RTA spiders have lost hemocyanin subtypes a–f, but retained subtype g, with *D. jiaxiangi* forming a distinct lineage relative to aquatic and terrestrial spiders (Figure 5C). Importantly, intertidal *D. jiaxiangi* exhibits substantial lineage‐specific expansion, with 12 hemocyanin copies, compared with seven in the water spiders and six in *Cupiennius salei* (CUSA). This amplification is consistent with selection for enhanced oxygen transport or buffering capacity in chronically hypoxic intertidal environments.

### 3.10 Sensory System Adaptation via Chemoreceptor Expansion

To explore how sensory systems have co‐evolved with other adaptive traits, we investigated the composition and evolution of chemoreceptor gene families across representative spider genomes. Our analysis revealed substantial interspecific variation in the copy numbers of ionotropic receptors (*IRs*/*iGluRs*) and gustatory receptors (*GRs*).

*D. jiaxiangi* possesses 125 *GR* and 146 *IR* gene copies, with IRs exceeding GRs in abundance (Figure S10A). This pattern suggests a predominant role for ionotropic glutamate receptors for detecting environmental cues, including humidity, salinity, and possibly chemical signals associated with prey or mates. Notably, *D. jiaxiangi* retains *IR93a*, which clusters with *Drosophila melanogaster IR93a* (Figure S10C), along with ionotropic receptors *IR25a* and *IR40a*, receptors known to mediate humidity sensing in hydrosensory sacculus neurons (Croset et al. 2010). Collectively, *D. jiaxiangi* harbours a higher number of IRs than most other spider species examined, reflecting enhanced capacity for chemical and physical environmental sensing.

Comparisons across spider life tree revealed that basal and aquatic spider clades generally possess fewer chemosensory receptors than aerial web‐building spiders (Figure S10A). However, both *Dysdera silvatica* and *D. jiaxiangi* exhibit a higher abundance of IRs relative to GRs, a trend also observed in other species associated with soil or aquatic environments (Figure S10).

Phylogenetic reconstruction of GRs (Figure S10B) and IRs (Figure S10C) including *D. jiaxiangi* and *D. melanogaster* revealed contrasting evolutionary dynamics. *GRs* from *D. jiaxiangi* form distinct clades from those of *D. melanogaster* , indicating substantial lineage‐specific diversification. However, several *D. jiaxiangi IR* families, such as *IR8a*, *IR25a*, *IR40a* and *IR93a*, cluster with their insect counterparts, suggesting deep evolutionary conservation of these *IR* receptors across arthropods. These patterns are consistent with observations in *Dysdera silvatica* (Escuer et al. 2021).

In summary, these results indicate that the expansion and diversification of chemoreceptor gene families in *D. jiaxiangi* highlights evolutionary fine‐tuning of sensory systems to meet the demands of a highly variable intertidal environment.

## 4 Discussion

Although ecological and behavioural adaptations of *Desis* species have been well documented (Leggett et al. 2024), the genomic basis under their remarkable intertidal lifestyle has remained largely unexplored. In this study, we present the first chromosome‐level genome of the obligate intertidal spider *D. jiaxiangi*, together with a high‐quality genome of the water spider *Argyroneta aquatica* (Figure 1). These resources provide a robust foundation for comparative multi‐omics analyses and allow us to identify key molecular innovations (Figure 5D) enabling spiders to thrive in their unique environments characterized by periodic submersion, hypoxia, salinity fluctuation and intense ultraviolet radiation.

Before considering the broader implications of these findings, it is important to explicitly acknowledge several limitations. Functional validation of the identified candidate genes was not conducted due to the technical challenges associated with the heterologous expression of large spidroins and the lack of established genetic or cell line tools for this non‐model species. Consequently, our interpretations are based on convergent genomic, structural and comparative evidence and should be viewed as hypotheses that motivate future functional work.

### 4.1 Climate‐Driven Demographic Dynamics in an Intertidal Specialist

Demographic reconstruction of *D. jiaxiangi* suggests that its demographic history closely tracks Pleistocene sea‐level oscillations (Figure 1C). Notably, effective population size peaked during periods of sea‐level low stand, when expanded intertidal habitats likely provided increased ecological opportunity, followed by a pronounced decline beginning around 100 ka—coinciding with the onset of the Last Glacial Period (Lambeck and Chappell 2001). Global cooling and climatic instability during this interval (Yokoyama et al. 2018) likely induced suitable habitats and prey availability, imposing demographic bottlenecks on *D. jiaxiangi* and potentially on other coastal arthropods. These results suggest that intertidal specialization confers both ecological opportunity and demographic vulnerability, with population size tightly coupled to shoreline dynamics.

### 4.2 Genomic Adaptations to Wave Exposure and UV Stress

The intertidal zone imposes intense mechanical and radiation stress, and our comparative genomic analyses reveal signatures consistent with adaptation to these challenges. *D. jiaxiangi* shows a marked expansion of genes involved in hormone biosynthesis and DNA repair. In particular, the expansion of *farnesol dehydrogenase* (*FDH*), a key enzyme in juvenile hormone biosynthesis (Kumar et al. 2022), may facilitate flexible regulation of developmental and reproduction in response to tidal rhythms and environmental unpredictability. The markedly higher copy number of *FDH* (12 copies) in *D. jiaxiangi* compared with both terrestrial and aquatic spiders (5 in *Pardosa pseudoannulata* and just 1 in *A. aquatica* (Figure S4D)) supports this interpretation.

In parallel, the expansion of DNA repair gene *AlkB*, known to reverse UV‐induced alkylated damage (Yang et al. 2008), suggests enhanced capacity to maintain genome integrity under prolonged exposure to solar radiation during low tides. Together, these patterns suggest that *D. jiaxiangi* has evolved a robust genomic toolkit that buffers both physiological and genetic stability against the combined effects of wave action and UV stress.

### 4.3 Osmoregulatory Evolution Under Fluctuating Salinity

Intertidal spiders experience rapid and repeated shifts in salinity arising from tidal inundation, evaporation and prey digestion (Leggett et al. 2024). We identified signatures of adaptive evolution in the β‐subunit of Na^+^/K^+^‐ATPase (*ATP1B*, Figure 2A,B), a core component of ionic homeostasis that stabilizes sodium and potassium gradients across cell membranes (Morth et al. 2007). Structural modifications in ATP1B may enhance ion transport efficiency or regulatory flexibility, enabling *D. jiaxiangi* to maintain osmotic balance across highly salt‐variable external conditions. These results parallel osmoregulatory adaptations reported in other intertidal and euryhaline taxa, underscoring convergent solutions to salinity stress.

### 4.4 Chemosensory Adaptation in a Chemically Complex Habitat

The intertidal zone presents a chemically heterogeneous landscape shaped by seawater, decaying organic matter, and diverse prey assemblages (Marlene A. Leggett et al. 2024). Our analyses reveal signatures of positive selection in odorant‐binding protein (OBP), key mediators of olfactory (Qu et al. 2021), suggesting enhanced chemosensory discrimination relevant to foraging, predator avoidance, and habitat selection.

More broadly, *D. jiaxiangi* exhibits substantial expansion and diversification of chemosensory receptors, particularly ionotropic receptors (IRs/iGluRs), relative to gustatory receptors (GRs) (Vizueta et al. 2018). Comparative analyses across 13 representative spider genomes indicate that this IR‐based expansion is shared with other species inhabiting soil‐associated or aquatic environments, consistent with a heightened reliance on chemical and physical environmental cues. The retention and expansion of conserved IRs involved in humidity sensing (e.g., IR25a, IR40a, IR93a) further suggest that chemosensory systems in *D. jiaxiangi* are finely tuned to detect microclimatic fluctuations that signal tidal transitions or suitable refuges. Together, these findings point to sensory system remodelling as a key component of intertidal adaption.

### 4.5 Molecular and Structural Innovation of Silk for Underwater Performance

While terrestrial spider silks have been extensively studied for their exceptional strength and elasticity (Vollrath and Knight 2001; Zhang et al. 2019), the properties and mechanisms of intertidal silk remain poorly understood. Proteomic analysis of *D. jiaxiangi* silk identified five distinct spidroin types, including a specialized Sp spidroin, which appears critical for underwater retreat construction. Although spidroin diversity may be underrepresented due to sampling limitations, the consistent detection of Sp spidroin supports its functional specialization in the intertidal environment.

Notably, Sp spidroins of aquatic‐associated spiders are enriched in glycine‐valine (GV) repeat regions, a sequence hallmark previously linked to hydrophobicity and underwater silk performance (Correa‐Garhwal et al. 2019). Among examined taxa, *D. jiaxiangi* shows the highest GV repeat count (74 repeats). Consistent with this molecular signature, contact angle measurements exceeding 90° for both ultrapure water and seawater confirm the intrinsic hydrophobic nature of *D. jiaxiangi* silk fibres (Law 2014) (Figure 4A,B).

Low‐complexity domains containing GVGAKV motifs were distributed throughout the Sp spidroin sequence in *D. jiaxiangi* (Figure 4H–J). AlphaFold2 predictions suggest that these GV‐rich regions adopt amyloid‐like 3D structures, characterized by reduced α‐helices and increased solvent‐exposed residues (Figure 4I,K, Figure S9E). Although amyloid‐like interactions have been implicated in silk assembly (Stewart and Stringer 2012), these predictions primarily indicate enhanced β‐sheet propensity and the potential for extensive intra‐ and intermolecular interactions. The interspersion of GV‐rich motifs with KV residues, known contributors to β‐sheet formation in thermoresponsive hydrogels (Löwik et al. 2010), may balance hydrophilicity with solubility and molecular alignment during fibre spinning (Jin and Kaplan 2003; Pochan et al. 2003; Rising and Johansson 2015). Together, these features promote efficient fibre assembly and mechanical resilience under dynamic aquatic conditions.

Beyond sequence composition, the physical morphology of intertidal spider silk further enhances its water‐repellent properties. *D. jiaxiangi* silk shows noncircular cross‐sections and substantial nanoscale diameter variation, ranging from 1 to 2 μm down to ~50 nm (Figure 4F), suggesting the integration of multiple silk types within the web. Such structural heterogeneity, combined with fine surface topography and micrometre‐scale mesh gaps, likely enhances hydrophobicity and water repellency (Öner and McCarthy 2000). The 3D‐mesh architecture generates high Laplace pressure (Equation 1), preventing seawater infiltration even under submersion (García‐Payo et al. 2000):

(1) $$∆p=-\gamma \left(\frac{1}{R_{1}}+\frac{1}{R_{2}}\right)$$

where ∆p is the Laplace pressure, γ is the surface tension of water and *R*_1_ and *R*_2_ are principal radii of curvature.

Although major ampullate silk of terrestrial spiders is coated with lipids and glycoproteins that provide environmental protection (Sponner et al. 2007), we found that the terrestrial funnel weaver sheet webs (e.g., *Allagelena difficilis* )—despite possessing surface lipids—retain hydrophilic properties (contact angle < 90°, Figure 4L). In contrast, terrestrial spider egg sacs are universally hydrophobic, reflecting their role in protecting offspring from moisture.

The silk retreats of *D. jiaxiangi* exhibit hydrophobicity comparable to terrestrial egg sacs rather than typical terrestrial capture webs. We therefore propose that *D. jiaxiangi* has evolved a molecular strategy—via specific hydrophobic spidroin motifs—to achieve ‘egg‐sac‐like’ intrinsic hydrophobicity in its silk retreat. This adaptation likely ensures long‐term stability of the air chamber under hydrostatic pressure, a function that surface lipids alone—prone to disruption by flow and abrasion—would be insufficient to maintain. The convergence between aquatic silk retreats and terrestrial egg sacs highlights the evolutionary recruitment of conserved protein motifs to meet the demands of underwater survival.

### 4.6 Silk as a Putative Respiration Interface in the Intertidal Zone

Remarkably, the silk retreats of *D. jiaxiangi* appear to function not only as physical refugia but also as components of an integrated respiration strategy. We detected hemocyanin—a copper‐based respiratory protein—within the silk proteome, suggesting that hemolymph‐derived components may be deposited onto silk to facilitate oxygen retention within submerged silk retreats.

Hemocyanin is a large, multi‐domain protein (630–660 amino acids) containing six copper‐binding active sites that reversibly bind oxygen in response to ambient concentrations (Gaykema et al. 1984; Volbeda and Hol 1989). In arthropods, hemocyanins are multifunctional, contributing to respiration, osmoregulation, cuticle synthesis and immune defence (Perry et al. 2019) and are typically expressed in the abdomen, legs and venom glands (Figure S8). Such expression patterns are consistent with a crucial role in oxygen transport, particularly when book lungs are immersed and direct gas exchange is compromised (Rehm et al. 2012; Riciluca et al. 2020).

Unlike horseshoe crabs, which rely on specialized book gills for direct seawater respiration, spiders possess book lungs adapted for aerial gas exchange and are poorly suited for direct function in seawater. Intertidal spiders must therefore adopt alternative respiratory strategies during submersion. Elevated hemocyanin expression in the abdomen and venom glands likely enhances internal oxygen transport (Rehm et al. 2012) during tidal inundation, when external exchange is severely restricted.

In *D. jiaxiangi*, the presence of hemocyanin in silk proteome raises the intriguing possibility that hemolymph proteins are secreted onto silk fibres, contributing to localized oxygen regulation within submerged silk retreats. Similar mechanisms have been proposed for underwater webs of *A. aquatica* and the egg sacs of semi‐aquatic spider *Dolomedes triton* (Correa‐Garhwal et al. 2019). Although in vitro oxygen‐binding activity of silk‐associated hemocyanin remains to be experimentally verified, its repeated detection—eight hemocyanin entries compared with four spidroin entries (Table S22)—and its known secretion from silk glands (Chaw et al. 2015; dos Santos‐Pinto et al. 2019) support its functional integration into the respiratory microenvironment of intertidal spiders. Ecologically, this integration may be critical during high tide, when the silk retreat functions as both a physical barrier and a biochemical interface for oxygen management under hypoxic underwater conditions.

Comparative genomic analysis further revealed extensive hemocyanin diversification in *D. jiaxiangi*, which possesses 12 hemocyanin genes arranged into two distinct types. By contrast, the horseshoe crab *Limulus polyphemus* harbours only seven hemocyanin genes (NCBI RefSeq assembly GCF_000517525.1), despite having larger multimeric hemocyanin complexes (8 × 6‐mers), whereas spiders of the RTA clade typically form 2 × 6‐mer hemocyanin (Rehm et al. 2012). Typical hemocyanin proteins contain six active sites, each coordinating two copper ions for reversible oxygen binding. Intriguingly, *D. jiaxiangi* encodes several truncated hemocyanin variants—some approximately half the length of canonical hemocyanin and retaining only one or three active sites—likely arising from partial gene duplications. These truncated forms may expand functional diversity and provide physiological flexibility under fluctuating oxygen conditions associated with tidal cycling.

The tight genomic clustering of hemocyanin genes in *D. jiaxiangi* likely facilitates ‘Gene Dosage Control’ and ‘Co‐regulation’ (Hurst et al. 2004; Spitz and Furlong 2012), enabling rapid, synchronized transcriptional responses to sudden hypoxic stress. Because hemocyanin functions as a multi‐subunit complex, precise stoichiometric production of subunits is essential for proper holoprotein assembly. From an ecological perspective, this compact genomic organization, together with the presence of truncated variants, may confer a selective advantage in the intertidal zone by allowing the entire locus to be simultaneously accessible to transcriptional machinery during abrupt environmental transitions, such as tidal submergence and the rapid onset of hypoxia.

Although in vitro functional validation of individual proteins (e.g., recombinant silk mechanics or ATPase activity) remains technically challenging in non‐model species, our differential expression analyses provide strong indirect evidence supporting the physiological relevance of these hemocyanin variants and other candidate adaptive genes (Figure 3D).

### 4.7 Metabolic and Sensory Tuning Across Tidal Cycles

Survival in the intertidal zone requires extreme metabolic plasticity: metabolic suppression during submergence followed by rapid activation during a brief low‐tide foraging window. Our results identify hexokinase (*GCK*) as a pivotal node in this transition. The combination of positive selection and environmentally responsive expression suggests evolutionary ‘tuning’ of GCK to support high‐flux glycolysis (Lin and Xu 2016), enabling *Desis* spiders to rapidly transition from quiescence to intense predatory activity upon emersion.

Conversely, the upregulation of *KLF15* and *NMRK1* during submergence indicates an active, gene‐regulated entry into hypometabolism rather than simple passive asphyxiation. *KLF15* coordinates fasting response and suppresses anabolic energy expenditure (Jeyaraj et al. 2012), while *NMRK1* maintains NAD^+^ homeostasis essential for basal cellular survival under hypoxia. This intensified selection on *ESRRB* further suggests active maintenance of mitochondrial integrity, mitigating oxidative stress during re‐oxygenation.

The relaxation of selection on visual pathways, coupled with retention of mechanosensory genes, aligns with the ecological constraints of *Desis*. Living deep within crevices or sealed retreats, visual acuity offers limited benefit, whereas mechanosensation (vibration detection via trichobothria) remains critical for detecting prey, wave action and tidal rhythms. Interestingly, upregulation of *GABRA6‐like* (GABA receptor) during active phases suggests enhanced inhibitory signaling, potentially acting as a sensory filter that suppresses background hydrodynamic noise while preserving salient prey cues immediately upon air exposure.

Consistent with this ecological framework, *Sp spidroin* expression is upregulated during aerial exposure, reflecting increased demands for web maintenance, retreat reinforcement, and prey capture. These transcriptomic patterns (Figure 5D) reinforce the functional roles inferred from genomic and proteomic analyses and demonstrate how gene regulation dynamically tracks environmental transitions across the land‐sea interface.

### 4.8 Conclusions and Outlook

In summary (Figure 5D), our study provides a comprehensive molecular framework for understanding adaptations to intertidal environments in arthropods. By integrating chromosome‐scale genomics with transcriptomic and proteomic data, we reveal how *D. jiaxiangi* has evolved coordinated innovations in silk biophysics, respiration, osmoregulation, metabolism and sensory processing to occupy one of the most physically demanding ecological niches on Earth.

Although functional validation remains constrained by technical limitations, our identification of candidate adaptive loci—including genes involved in silk structure, DNA repair, oxygen transport and metabolic regulation—provides a robust framework for future functional studies. Beyond evolutionary insights, the molecular strategies uncovered here provide promising blueprints for bioinspired material science, particularly for the design of next‐generation fibres and coatings capable of functioning in wet, saline and corrosive environments.

## Author Contributions

D.L., Q.S. and W.Z. contributed to the conceptualization and funding acquisition of the project. F.L., Y.L., Y.Z., Q.M.P., C.B., Y.W., L.C., L.Y. and S.G., contributed to the methodology, investigation, data collection and visualization. The writing was led by F.L., D.L., Q.S., W.Z., Y.L., Q.M.P. and Y.Z. with reviewing, revising and editing. All authors have read and approved the manuscript for publication.

## Conflicts of Interest

The authors declare no conflicts of interest.

## Supporting information


**Figure S1:** Sampling *D. jiaxiangi* in an intertidal zone of Hainan Island, China.

**Figure S2:** A 17‐mer analysis of the *D. jiaxiangi* genome.

**Figure S3:** The genome‐wide Hi‐C heatmaps of *D. jiaxiangi* (A) and *A. aquatica* (B).

**Figure S4:** GO and KEGG enrichment analyses of expanded gene families in the *Desis jiaxiangi* genome identified by cafe5. (A) Phylogeny of representative spider species. Numbers on each node indicate gene family expansions and contractions. The star indicates the foreground branch used in the branch‐site model test. (B) Significantly enriched KEGG pathways (adjusted *p* < 0.05). (C) Significantly enriched GO terms (adjusted *p* < 0.001). (D) Unrooted gene tree of the significantly expanded insect hormone biosynthesis gene *farnesol dehydrogenase*. (E) Unrooted gene tree of the significantly expanded DNA repair and recombination gene *AlkB*. (F) Unrooted gene tree of the significantly expanded p53 signalling pathway gene *RRM2*. Bule‐highlighted regions indicate *D. jiaxiangi*‐species branches. Species abbreviations: DYSI, *Dysdera silvatica* ; OEGI, *Oedothorax gibbosus* ; TRCL, *Trichonephila clavata* ; TRAN, *Trichonephila antipodiana* ; ARBR, *Argiope bruennichi* ; PATE, *Parasteatoda tepidariorum* ; PAPS, *Pardosa pseudoannulata* ; DEJI, *Desis jiaxiangi*; ARAQ, *Argyroneta aquatica* .

**Figure S5:** Identification and functional enrichment of genes under selection inferred from PAML and Hyphy analyses. (A) Numbers of significant genes detected by PAML and Hyphy, respectively. (B) Volcano plot showing the distribution of K values (reflecting shifts in selection pressure) for genes analysed using the RELAX model in Hyphy. (C) Gene Ontology (GO) enrichment analysis of rapidly evolving genes and genes under intensified selection.

**Figure S6:** KEGG enrichment analyses of gene sets identified under different selection models. Gene sets include rapidly evolving genes (PAML), positive selection genes (PAML and Hyphy BUSTED), genes supported by BEB posterior probability ≥ 0.95 (PAML), and genes under intensified or relaxed selection (Hyphy RELAX). Except for the BEB‐supported gene set (*p* < 0.05), all other enrichments were filtered using a false discovery rate threshold of *q* < 0.05.

**Figure S7:** Phylogeny, positively selected sites and protein structures of positively selected genes (PSGs) in three spider species. (A) ML tree, sequence alignment with positively selected sites of RHOGDI. (B) Predicted protein structures of RHOGDI. (C) ML tree, sequence alignment with positively selected sites of CAPON. (D) Predicted protein structures of CAPON. Full gene IDs for each specific protein are listed after *Desis jiaxiangi*. Specifically, RHOGDI corresponds to *DEJI_Chr16_g17865* and CAPON to *DEJI_Chr16_g17983*.

**Figure S8:** Top 50 expressed genes across tissues of the intertidal spider *D. jiaxiangi*. Shown are the most highly expressed genes in the abdomen, venom glands, book lungs, legs and the whole silk gland complex.

**Figure S9:** Structure prediction of *D. jiaxiangi* Sp spidroin. (A, B) Predicted dimer structures using AlphaFold2. Red squares indicate intradimer expected position error. (A) N‐terminal (NT) and C‐terminal (CT) domains dimers. (B) GV‐rich and ‘RAK’ domains dimers. (C–F) Predicted secondary structure, ordered regions and solvent accessibility using RaptorX. (C, E) GV‐rich domains; (D, F) ‘RAK’ domains. (G–H) Sequence similarity within the protein for the six GV‐rich domains and five ‘RAK’ domains. GVGAKV‐repeat motifs are highlighted. Dots indicate amino acid identity, and dashes indicate gaps or missing residues.

**Figure S10:** Chemoreceptor gene families in representative spider genomes. (A) Total number of chemoreceptor genes identified across 13 representative spider species. (B) Phylogenetic relationships among gustatory receptors in *D. jiaxiangi* and *Drosophila melanogaster* . (C) Phylogenetic relationships among ionotropic glutamate receptors in *D. jiaxiangi*, *Atypus karschi* , *Luthela beijing* and *Drosophila melanogaster* . Sequences from *D. jiaxiangi* are shown in blue, and sequences from *D. melanogaster* are shown in black. Pink circles indicate bootstrap support > 80%, and branch lengths correspond to substitution rates.

**Data S1:** men70147‐sup‐0011‐TableS1‐S26.xlsx.

## Data Availability

All data are available in the main text or the Supporting Information. Genome sequence and RNA‐seq data have been deposited in NCBI SRA (Bioprojects: PRJNA1167885 and PRJNA1183626) with accession codes from SRR30858880 to SRR30858896, SRR32660275 to SRR32660277, SRR36929850 to SRR36929851. The finalized chromosome‐scale genome assemblies have been deposited in NCBI/GenBank under the accession numbers (JBLXXJ000000000 for *Desis jiaxiangi* and JBJCYU000000000 for *Argyroneta aquatica* ). The gene annotation files (GFF3) are deposited in figshare (https://figshare.com/s/dc3df181dfec49e8334c). The mass spectrometry proteomics data has been deposited to the ProteomeXchange Consortium via the PRIDE (Perez‐Riverol et al. 2021) partner repository with the dataset identifier PXD057357. All protein structures predicted by the AlphaFold model are available in figshare (https://figshare.com/s/1ce590f4e439a490c705). The code used for data analysis in this study is described in Methods in accordance with the guidelines provided by the relevant bioinformatic softwares.
