# Supplementary figures and images for: Transition From Land to Sea: Comparative Genomics Illuminates the Adaptive Evolution of the Intertidal Spider
Source: Mol Ecol Resour. 2026 Apr 27;26:e70147. doi: 10.1111/1755-0998.70147 (PMC13111977; doi:10.1111/1755-0998.70147)

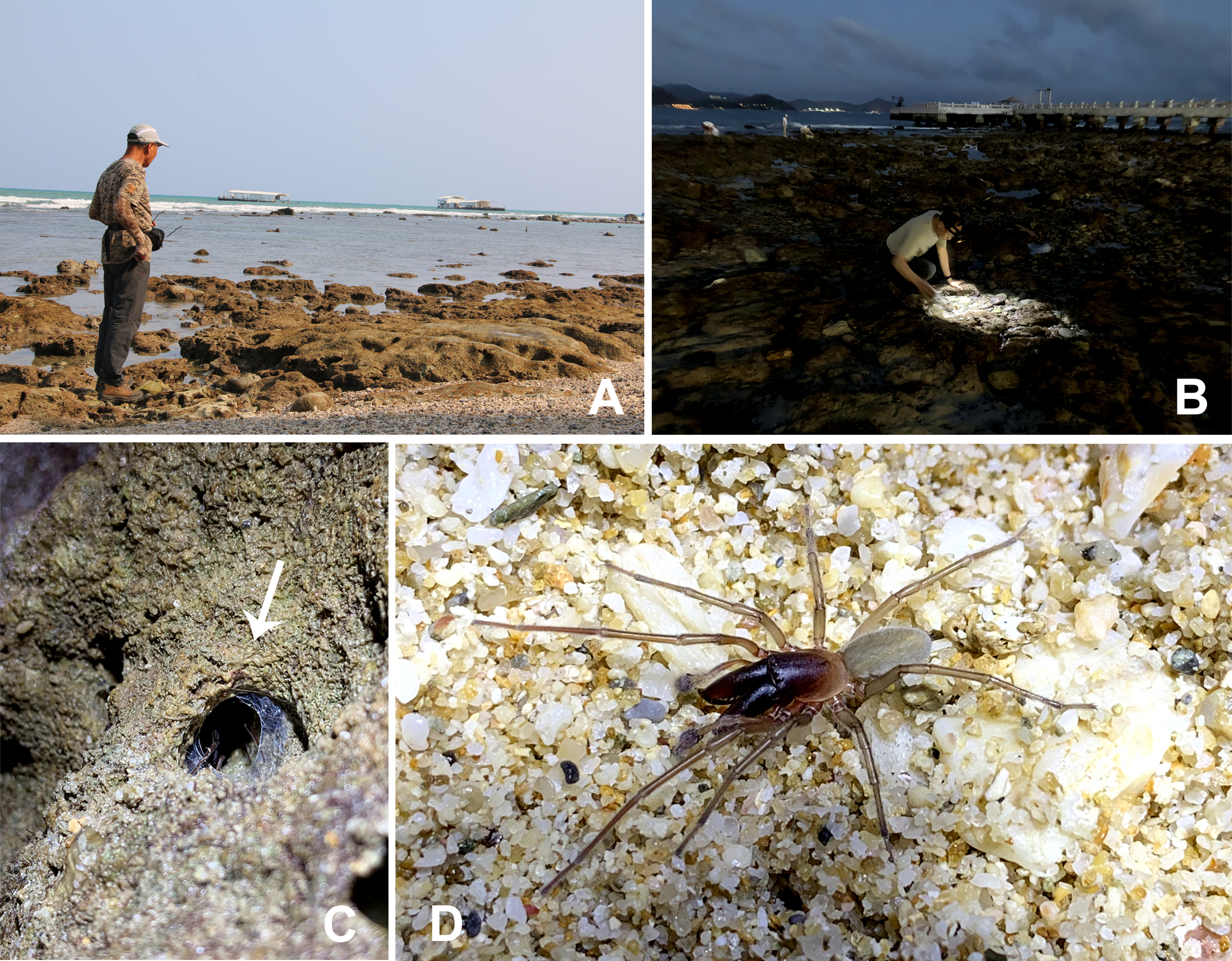

Supplement: Supplementary file 1 — Figure S1: Sampling D. jiaxiangi in an intertidal zone of Hainan Island, China. [file MEN-26-e70147-s009.jpg]

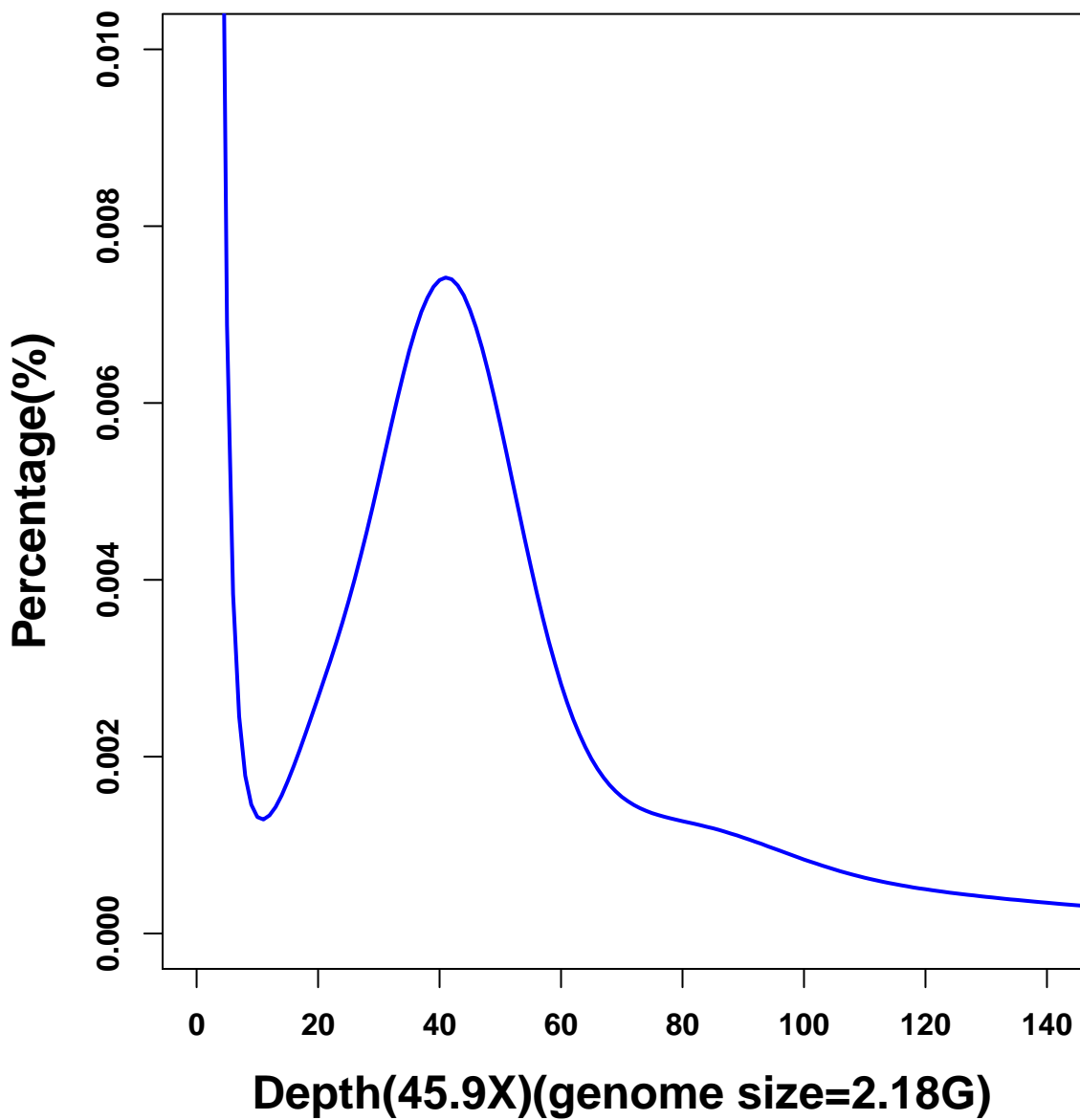

Supplement: Supplementary file 2 — Figure S2: A 17‐mer analysis of the D. jiaxiangi genome. [file MEN-26-e70147-s006.pdf]

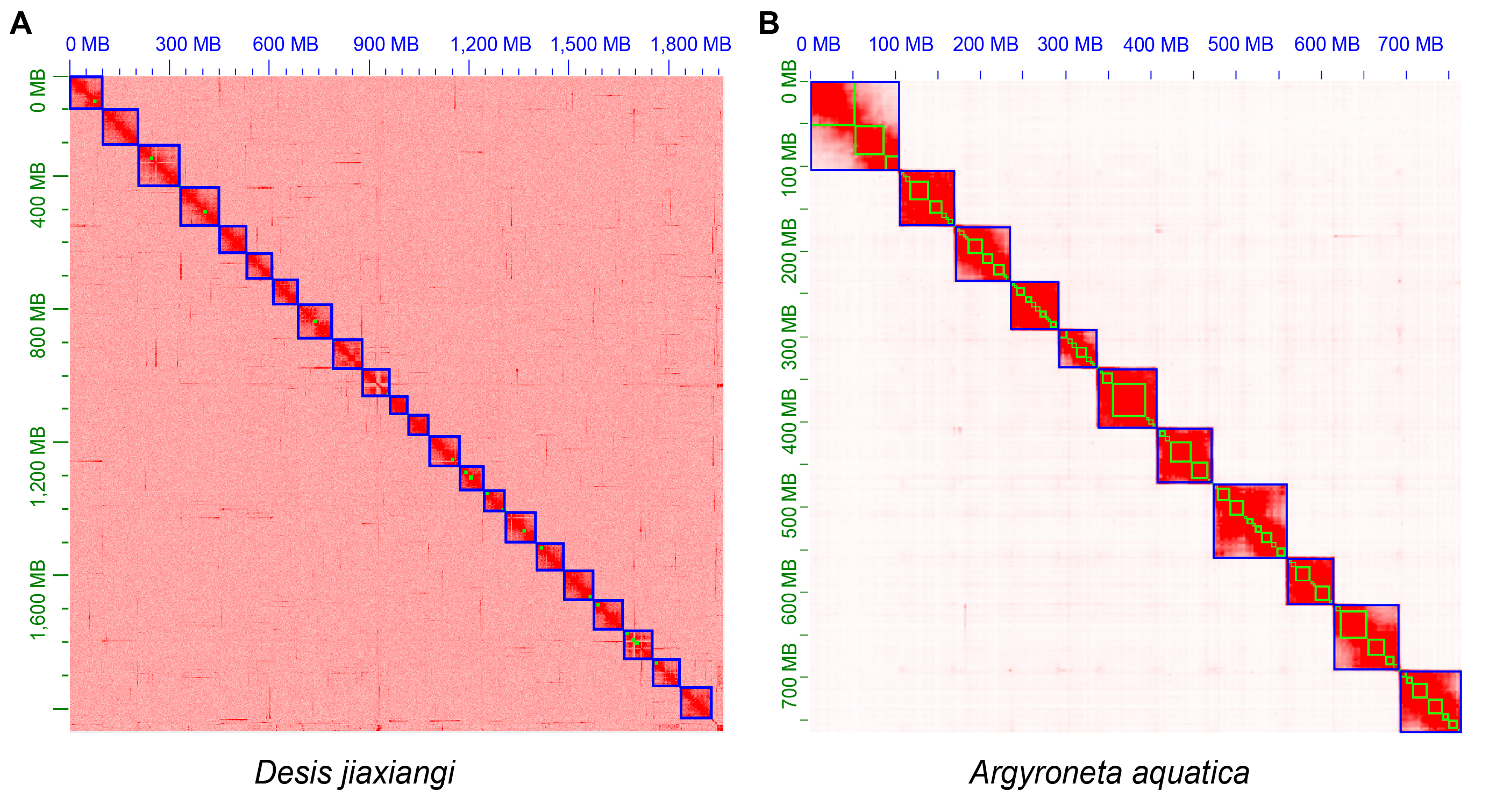

Supplement: Supplementary file 3 — Figure S3: The genome‐wide Hi‐C heatmaps of D. jiaxiangi (A) and A. aquatica (B). [file MEN-26-e70147-s001.png]

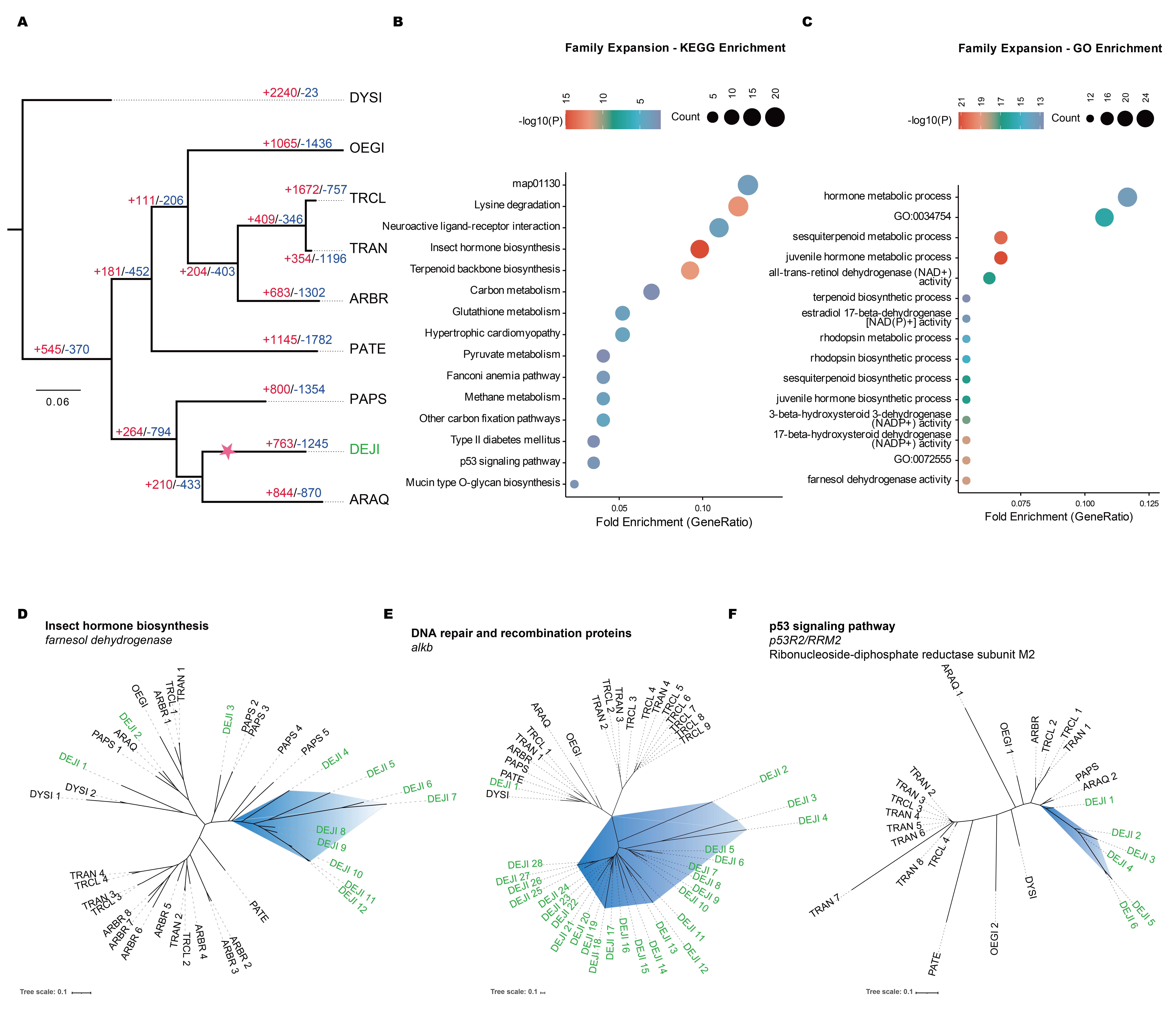

Supplement: Supplementary file 4 — Figure S4: GO and KEGG enrichment analyses of expanded gene families in the Desis jiaxiangi genome identified by cafe5. (A) Phylogeny of representative spider species. Numbers on each node indicate gene family expansions and contractions. The star indicates the foreground branch used in the branch‐site model test. (B) Significantly enriched KEGG pathways (adjusted p < 0.05). (C) Significantly enriched GO terms (adjusted p < 0.001). (D) Unrooted gene tree of the significantly expanded insect hormone biosynthesis gene farnesol dehydrogenase. (E) Unrooted gene tree of the significantly expanded DNA repair and recombination gene AlkB. (F) Unrooted gene tree of the significantly expanded p53 signalling pathway gene RRM2. Bule‐highlighted regions indicate D. jiaxiangi‐species branches. Species abbreviations: DYSI, Dysdera silvatica ; OEGI, Oedothorax gibbosus ; TRCL, Trichonephila clavata ; TRAN, Trichonephila antipodiana ; ARBR, Argiope bruennichi ; PATE, Parasteatoda tepidariorum ; PAPS, Pardosa pseudoannulata ; DEJI, Desis jiaxiangi; ARAQ, Argyroneta aquatica . [file MEN-26-e70147-s011.png]

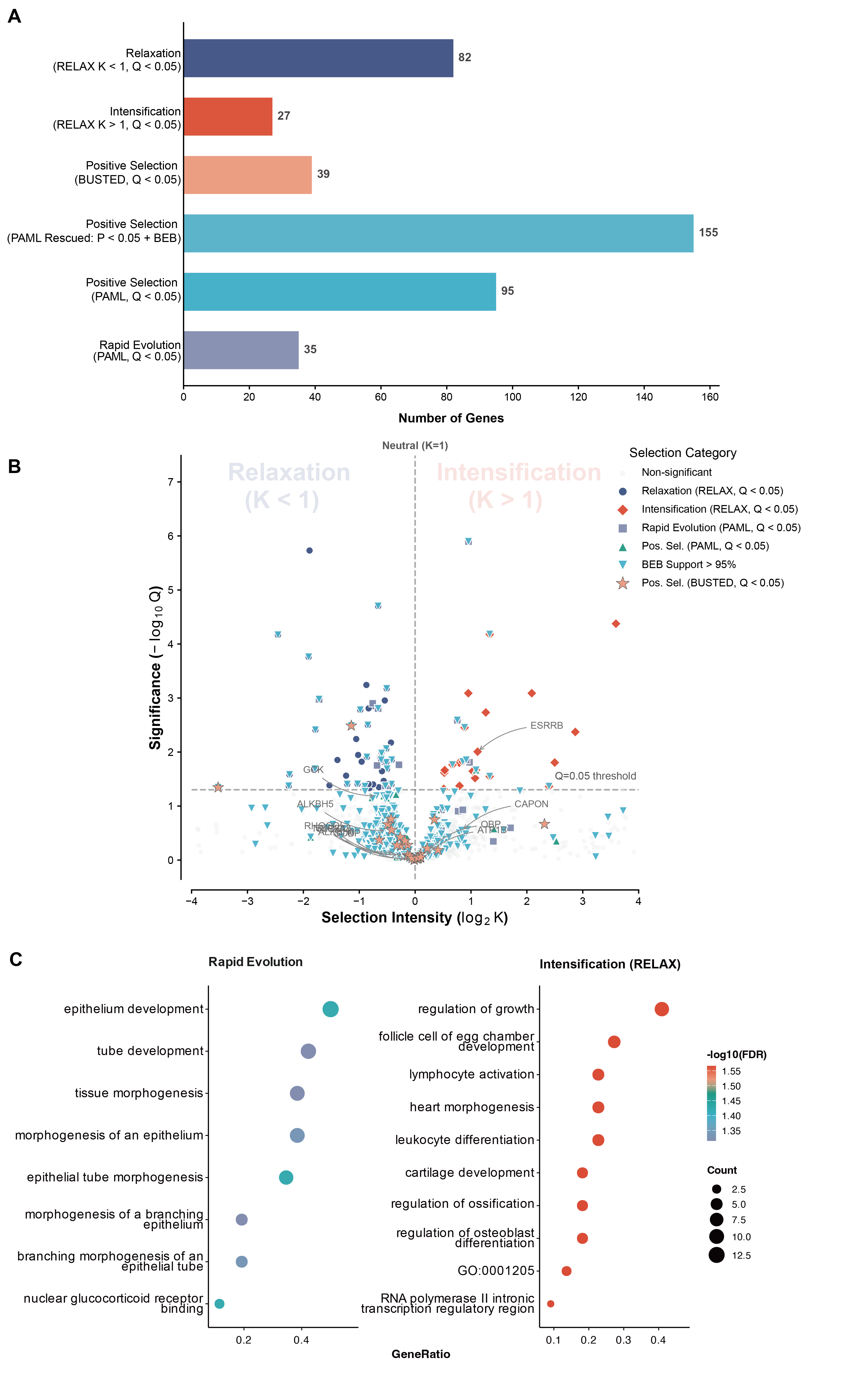

Supplement: Supplementary file 5 — Figure S5: Identification and functional enrichment of genes under selection inferred from PAML and Hyphy analyses. (A) Numbers of significant genes detected by PAML and Hyphy, respectively. (B) Volcano plot showing the distribution of K values (reflecting shifts in selection pressure) for genes analysed using the RELAX model in Hyphy. (C) Gene Ontology (GO) enrichment analysis of rapidly evolving genes and genes under intensified selection. [file MEN-26-e70147-s010.png]

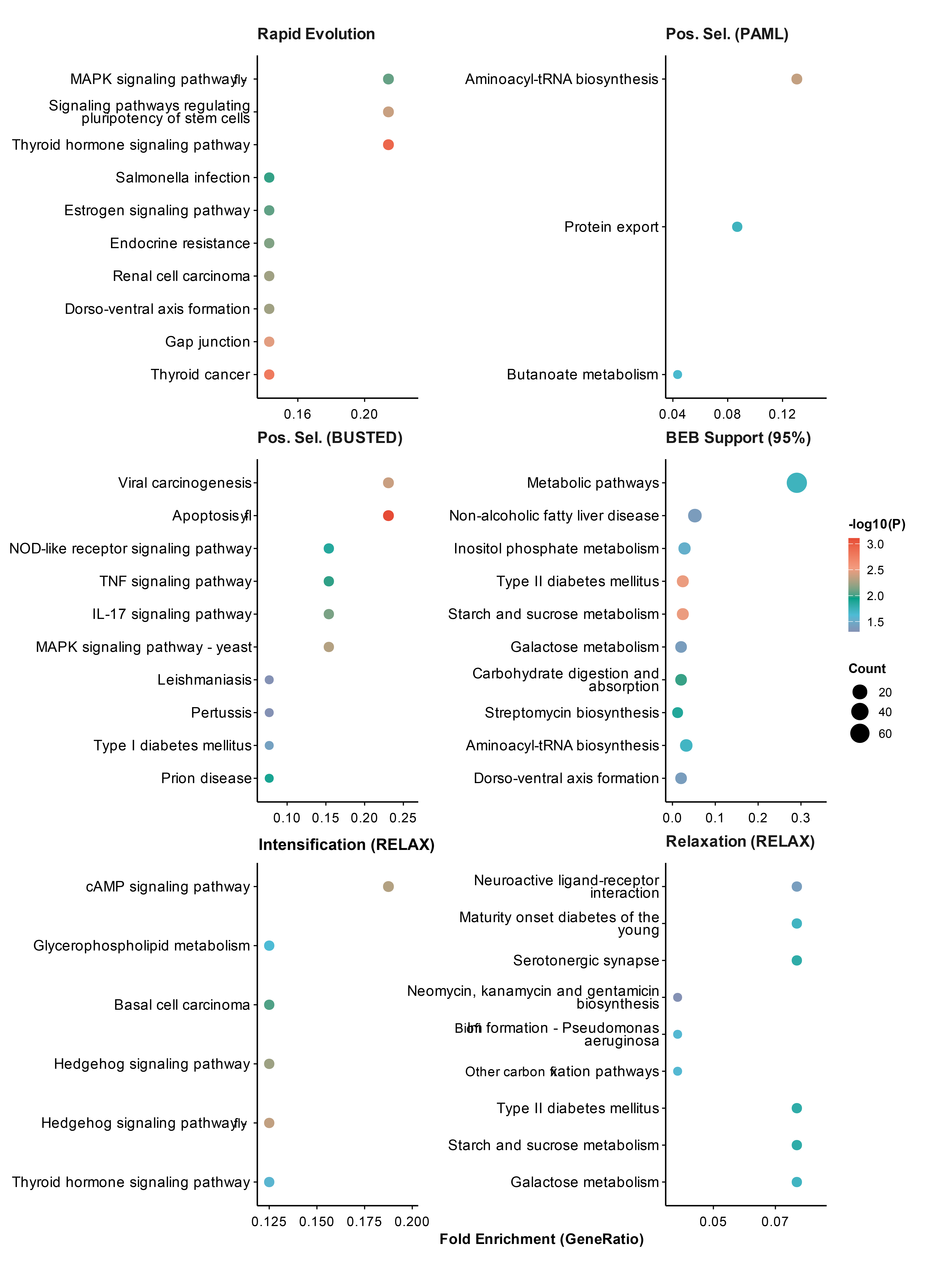

Supplement: Supplementary file 6 — Figure S6: KEGG enrichment analyses of gene sets identified under different selection models. Gene sets include rapidly evolving genes (PAML), positive selection genes (PAML and Hyphy BUSTED), genes supported by BEB posterior probability ≥ 0.95 (PAML), and genes under intensified or relaxed selection (Hyphy RELAX). Except for the BEB‐supported gene set (p < 0.05), all other enrichments were filtered using a false discovery rate threshold of q < 0.05. [file MEN-26-e70147-s007.png]

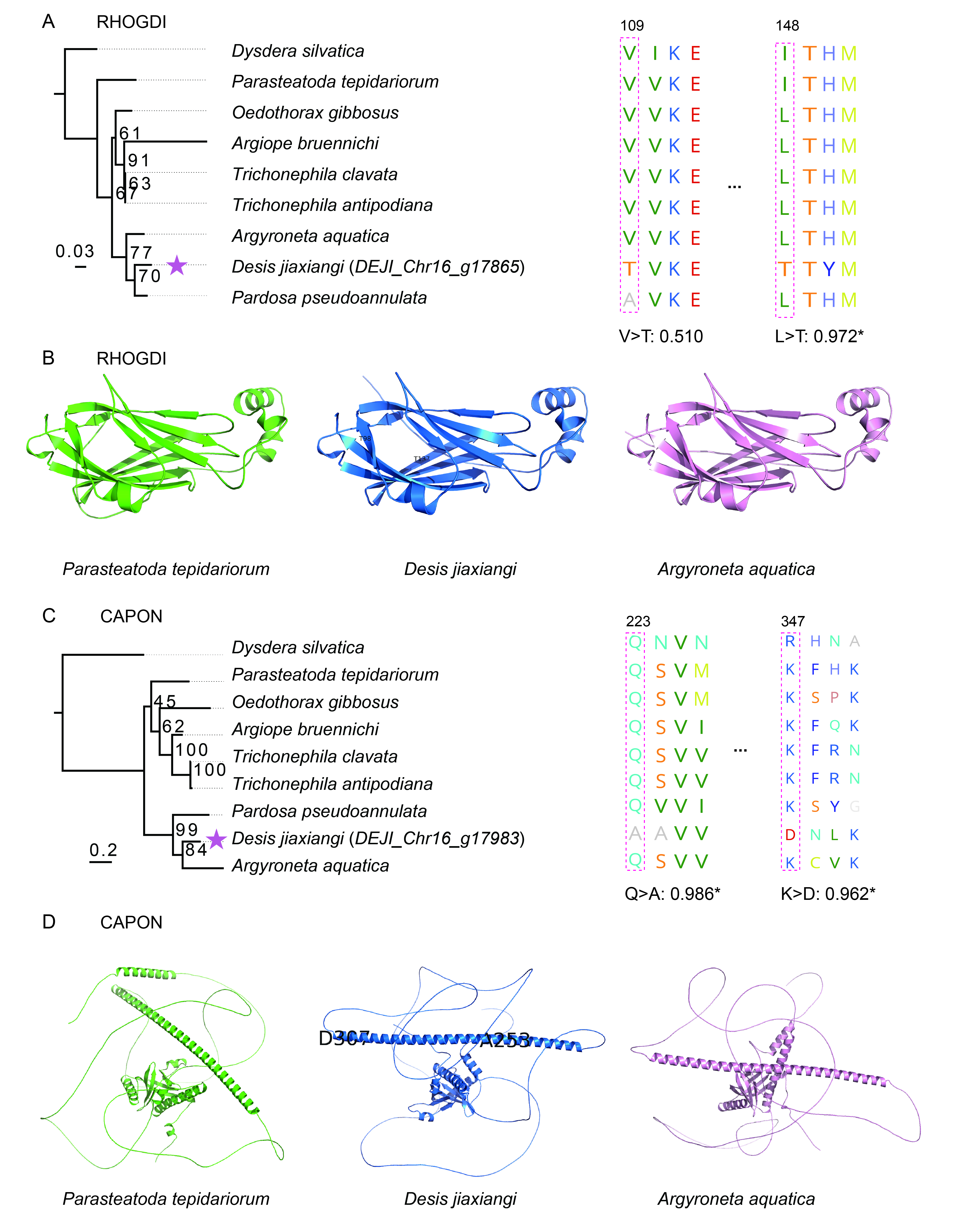

Supplement: Supplementary file 7 — Figure S7: Phylogeny, positively selected sites and protein structures of positively selected genes (PSGs) in three spider species. (A) ML tree, sequence alignment with positively selected sites of RHOGDI. (B) Predicted protein structures of RHOGDI. (C) ML tree, sequence alignment with positively selected sites of CAPON. (D) Predicted protein structures of CAPON. Full gene IDs for each specific protein are listed after Desis jiaxiangi. Specifically, RHOGDI corresponds to DEJI_Chr16_g17865 and CAPON to DEJI_Chr16_g17983. [file MEN-26-e70147-s005.jpg]

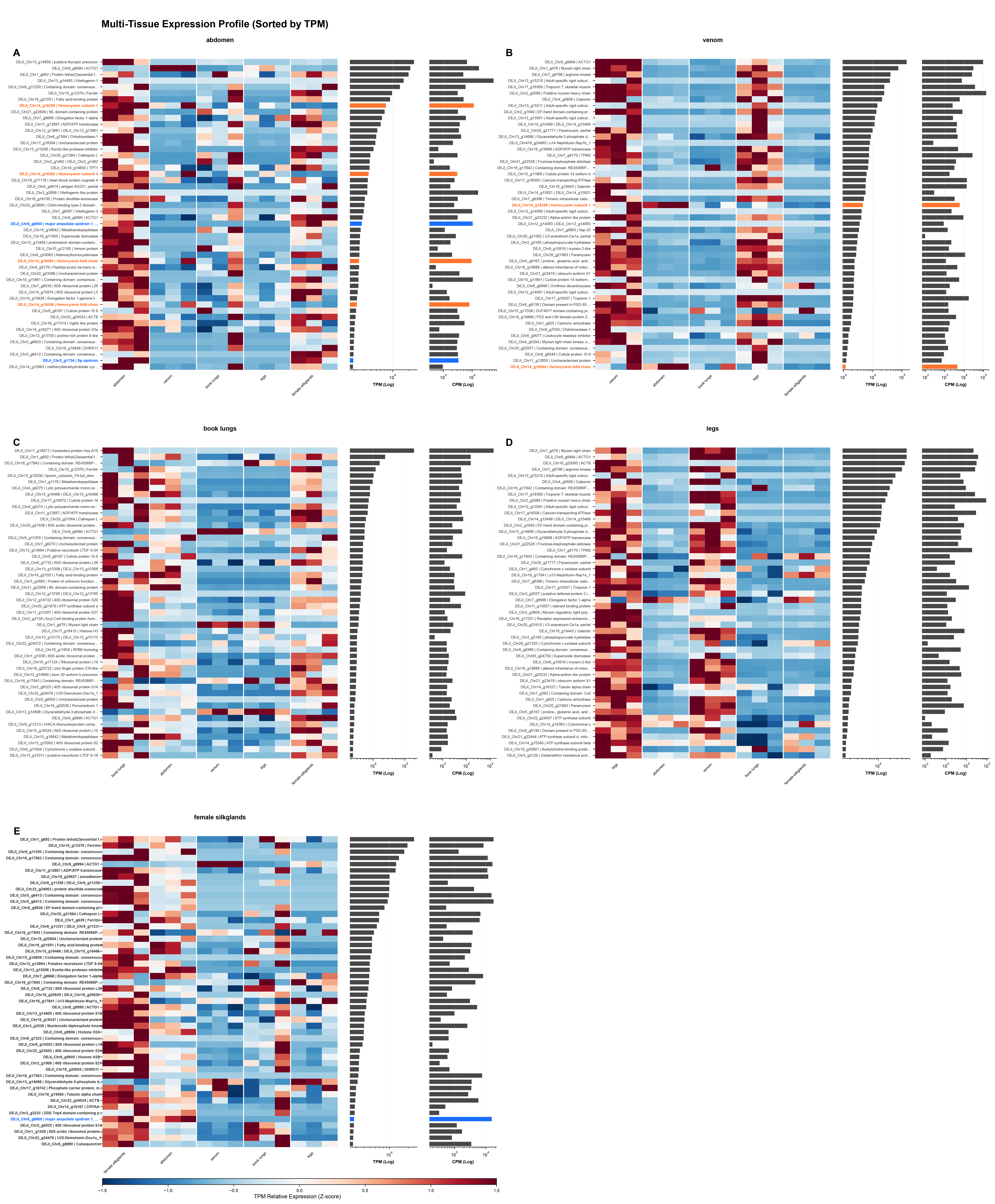

Supplement: Supplementary file 8 — Figure S8: Top 50 expressed genes across tissues of the intertidal spider D. jiaxiangi. Shown are the most highly expressed genes in the abdomen, venom glands, book lungs, legs and the whole silk gland complex. [file MEN-26-e70147-s002.png]

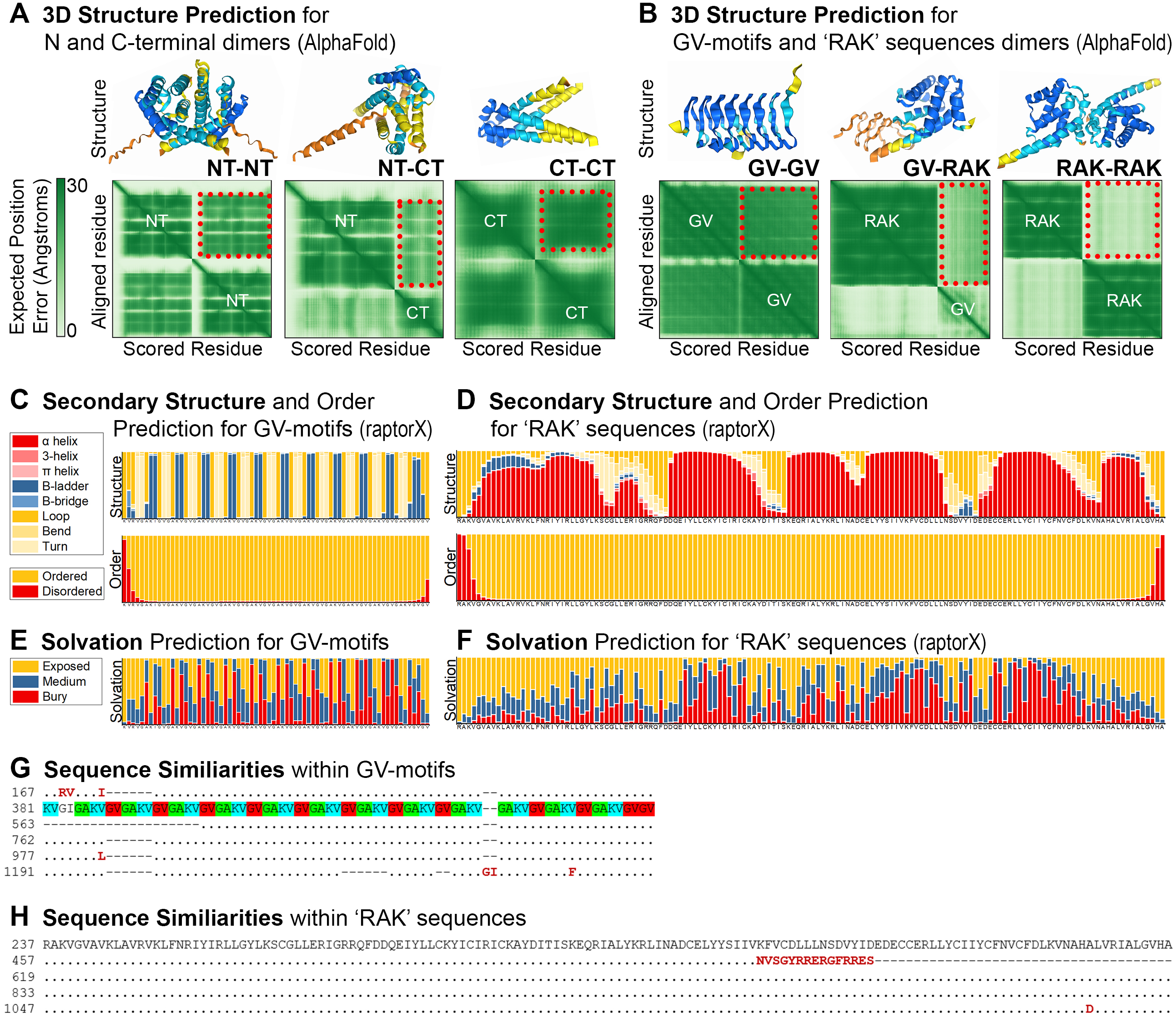

Supplement: Supplementary file 9 — Figure S9: Structure prediction of D. jiaxiangi Sp spidroin. (A, B) Predicted dimer structures using AlphaFold2. Red squares indicate intradimer expected position error. (A) N‐terminal (NT) and C‐terminal (CT) domains dimers. (B) GV‐rich and ‘RAK’ domains dimers. (C–F) Predicted secondary structure, ordered regions and solvent accessibility using RaptorX. (C, E) GV‐rich domains; (D, F) ‘RAK’ domains. (G–H) Sequence similarity within the protein for the six GV‐rich domains and five ‘RAK’ domains. GVGAKV‐repeat motifs are highlighted. Dots indicate amino acid identity, and dashes indicate gaps or missing residues. [file MEN-26-e70147-s008.jpg]

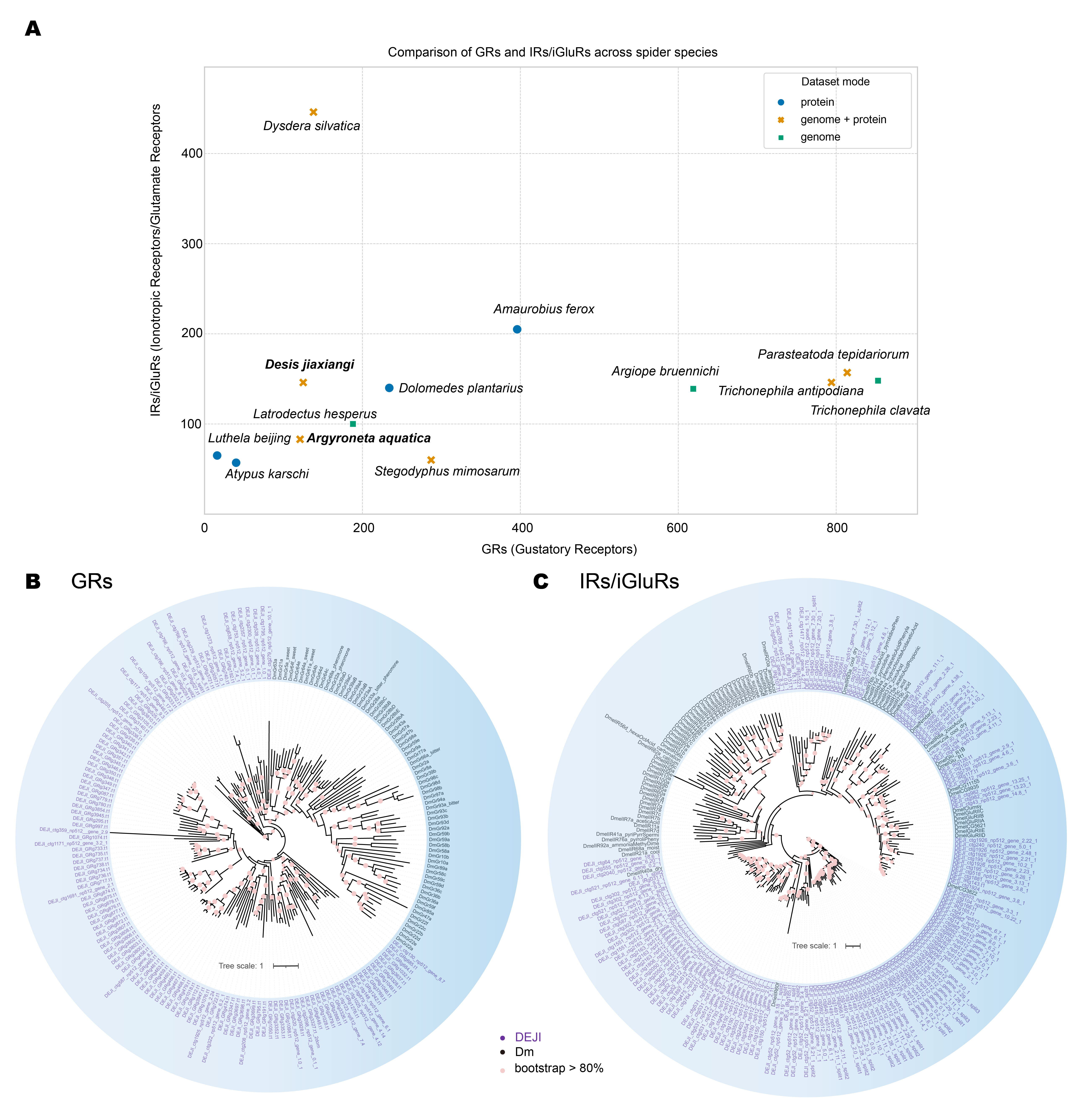

Supplement: Supplementary file 10 — Figure S10: Chemoreceptor gene families in representative spider genomes. (A) Total number of chemoreceptor genes identified across 13 representative spider species. (B) Phylogenetic relationships among gustatory receptors in D. jiaxiangi and Drosophila melanogaster . (C) Phylogenetic relationships among ionotropic glutamate receptors in D. jiaxiangi, Atypus karschi , Luthela beijing and Drosophila melanogaster . Sequences from D. jiaxiangi are shown in blue, and sequences from D. melanogaster are shown in black. Pink circles indicate bootstrap support > 80%, and branch lengths correspond to substitution rates. [file MEN-26-e70147-s003.jpg]
